# Cryo-EM reveals the membrane-binding phenomenon of EspB, a virulence factor of the mycobacterial type VII secretion system

**DOI:** 10.1016/j.jbc.2023.104589

**Published:** 2023-03-06

**Authors:** Nayanika Sengupta, Surekha Padmanaban, Somnath Dutta

**Affiliations:** Molecular Biophysics Unit, Indian Institute of Science, Bangalore, India

**Keywords:** EspB, phospholipid, membrane, ESX-1, T7SS, *mycobacterium*, transmission electron microscopy, cryo-EM

## Abstract

*Mycobacterium tuberculosis* (Mtb) utilizes sophisticated machinery called the type VII secretion system to translocate virulence factors across its complex lipid membrane. EspB, a ∼36 kDa secreted substrate of the ESX-1 apparatus, was shown to cause ESAT-6–independent host cell death. Despite the current wealth of high-resolution structural information of the ordered N-terminal domain, the mechanism of EspB-mediated virulence remains poorly characterized. Here, we document EspB interaction with phosphatidic acid (PA) and phosphatidylserine (PS) in the context of membranes, through a biophysical approach including transmission electron microscopy and cryo-EM. We were also able to show PA, PS-dependent conversion of monomers to oligomers at physiological pH. Our data suggest that EspB adheres to biological membranes with limited PA and PS. EM of yeast mitochondria with EspB indicates a mitochondrial membrane-binding property of this ESX-1 substrate. Further, we determined the 3D structures of EspB with and without PA and observed plausible stabilization of the low complexity C-terminal domain in the presence of PA. Collectively, our cryo-EM–based structural and functional studies of EspB provide further insight into the host–Mtb interaction.

*Mycobacterium tuberculosis*, a unique Gram-positive bacterium, is accounted as one of the most potent human pathogens. According to the latest reports form the World Health Organization, tuberculosis claimed a mammoth toll of nearly 1.5 million lives globally (1). A hallmark feature attributed to the virulence of this acid-fast bacterium is the unusual composition of its cell wall (2, 3, 4). An elaborate, highly hydrophobic capsule and a complex mycomembrane form an additional layer on top of the peptidoglycan and arabinogalactan matrix (5, 6, 7). This renders mycobacterial cells impervious to most drug molecules or therapeutic compounds (2, 5, 7, 8). However, in the forefront of determining pathogenic success is a multimachinery secretion apparatus, called the Type VII secretion system (T7SS), involved in promoting host cell death, modulation of immune response, zinc and iron assimilation, and maintaining cell wall integrity (5, 9, 10). Essential components of the T7SS include (i) transmembrane channel and associated membrane proteins that form the cardinal structural element, (ii) AAA+ or FtsK/SpoIIIE ATPases and chaperones that control substrate translocation, and (iii) substrates belonging to the PE/PPE (Pro-Glu/Pro-Pro-Glu) family or Esx proteins (9, 10, 11, 12, 13, 14, 15). Among the five homologous T7 systems, ESX-1 through ESX-5 (early secretory antigenic target [ESAT-6] secretion system 1–5), ESX-1 is the most well-studied system that is known to secrete at least five virulence associated factors—EsxA (ESAT-6), EsxB (CFP-10), EspB, EspC, and EspA (5, 9, 16, 17, 18). ESAT-6 and CFP-10 belong to the WxG100 family of secreted substrates and are encoded by the region of difference (RD1) locus of *M. tuberculosis* genome. It is the absence of this locus that renders compromised virulence in the vaccine strain *Bacillus* Calmette Guerin (9). Similar to the WxG100 family of heterodimeric complexes are the PE/PPE family heterodimeric substrates which have characteristic Pro-Glu(PE) or Pro-Pro-Glu(PPE) motifs in the N-terminal (19). EspB, which is exclusive to the ESX-1 system, is an exception for having the PE and PPE domains joined together by means of a flexible linker in a continuous polypeptide chain (20, 21). It is also the only example of a PE/PPE family secreted substrate that can oligomerize into higher order oligomers, predominantly heptamers (20, 22, 23). Oligomerization of EspB is observed only in the culture filtrate, that is, when the full-length EspB (1–460) is cleaved at the C-terminal domain by the subtilisin-type serine protease MycP_1_ (21).

EspB, denoted Rv3881c, is encoded by the extended RD1 region (extRD1) of *Mycobacterium tuberculosis* (24). Several *in vitro* and *in vivo* infection models have previously reported a close association of EspB with host cell toxicity, evasion of phagosome maturation, and extrapulmonary dispersal of tuberculosis (24, 25, 26, 27). It was shown that the secretion of EspB was unaffected by the cosecretion of other Esp secreted substrates EspA, EspC, and EspD (27). Provision of purified C-terminal–processed EspB to 5′ Tn:*pe35* mutant showed elevated levels of toxicity in THP-1 monocytes in a dose-dependent fashion (27). This gave rise to a notion that EspB elicits virulence in a manner distinct from EsxA. Crystal structure of monomeric EspB revealed a structured N-terminal domain comprising both WxG and YxxxD signal motif on the same side of a long coiled-coil helix bundle (20, 21). The C-terminal domain, however, remained unstructured due to high structural flexibility. This raises an interesting question—if MycP_1_ cleaves at the C-terminal domain after which the mature isoforms oligomerize—what could be the benefit of retaining ∼50 amino acid residue long stretch of disorder? Intriguingly, mature isoform of EspB is able to bind phospholipids such as phosphatidic acid (PA) and phosphatidylserine (PS), while full-length EspB does not show this property (27). Cryo-EM structure of full-length oligomeric EspB illustrated a heptameric ring-like assembly of the N-terminal domain (22). Surprisingly, it was observed that the inner diameter of EspB heptamer was nearly 4.5 nm, which indicated that the central channel was sufficiently large to transport other heterodimeric ESX-1 substrates such as ESAT6-CFP10 (22). This notion was further supported when more recently, a 7 + 1 model of EspB monomer trapped within a heptameric EspB channel was proposed for EspB_2–348_ (23). The N-terminal domain structure of EspB_1–460_ and its isoforms has been extensively characterized; however, to date, our understanding of the functional role of EspB and its low-complexity C-terminal domain remains limited. Therefore, the molecular basis for EspB-mediated pathogenesis requires further investigation. Past seminal work by Chen *et al.*, 2013 (27), had shown preferential binding of mature EspB with phospholipids. Nevertheless, to date, structural elucidation of this affinity has been elusive. Thus, to provide further insight into the functional aspect of C-terminal–processed EspB, we designed a single particle cryo-EM–based approach to study EspB–phospholipid interaction in a near-native environment. In this current study, we performed various biochemical and biophysical experiments to characterize the oligomeric states of EspB in the presence and absence of lipid and lipid membrane. Our study provides insight into lipid-dependent oligomerization of monomeric EspB into the well-studied heptameric pore-like assembly. Additionally, we implemented transmission electron microscopy (TEM) imaging to visualize heptameric EspB interaction with yeast mitochondria and with liposomes, which mimic outer membrane of host mitochondria. Furthermore, interaction of EspB with various lipids were confirmed by microscale thermophoresis (MST), and membrane sedimentation assay was performed to show the membrane-binding function of EspB. Analogous to EspB, ESAT-6 also possesses an unstructured C-terminal domain, which was recently shown to mediate granuloma formation and phagosomal membrane damage (28). From our study, we have identified the postsecretion isoform of *Mycobacterium tuberculosis* ESX-1 substrate, EspB, as an additional membrane-binding virulence factor secreted through the T7SS. Here, we report that EspB binds to host membranes through affinity for anionic phospholipids like PA) PS, and phosphatidylinositol-4-phosphate (PIP4). The abundance of inositol phosphates in phagosomal membranes and affinity of EspB to PIP4 suggest that EspB may prevent phagosomal maturation by binding to inositol phosphates, PA, and PS in the phagosomal membrane. Further, we show that EspB also binds to mitochondrial outer membrane but does not function as a membranolytic virulence factor. Thus, we propose that the membrane-binding property of EspB may play an important role in the survival of *M. tuberculosis* within the host macrophages.

## Results

### Recombinant EspB_1–332_ exists as diverse oligomeric species in solution

**Figure 1:**
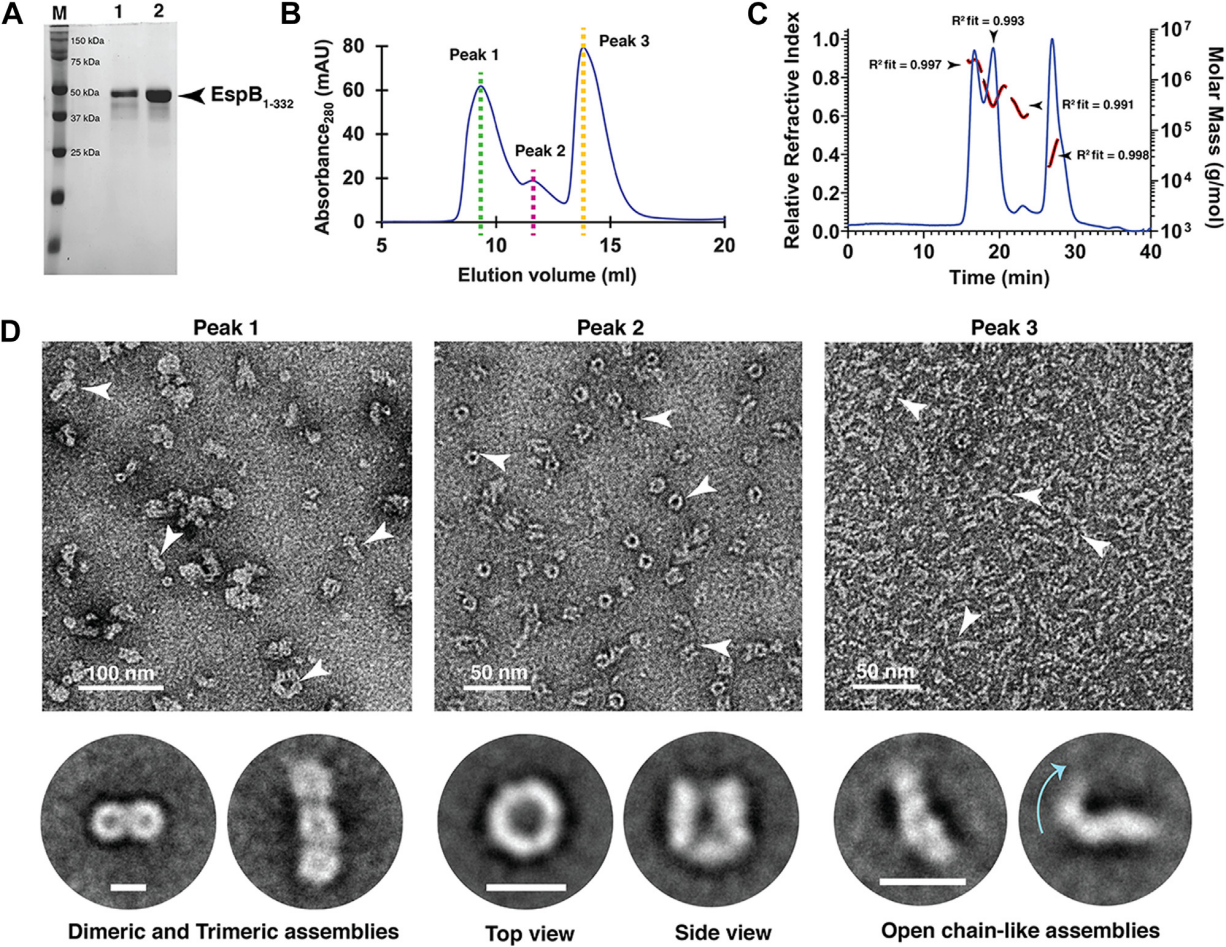
**Oligomeric states of purified EspB**_**1–332**_**.***A*, 12% SDS-PAGE showing the purity of recombinant EspB_1–332_. *B*, SEC elution profile shows three broad peaks corresponding to higher order oligomer and plausible monomer population. *Green* and *pink dashed lines* show the peak fractions of the high molecular weight assemblies (Peak 1 and Peak 2) of secreted EspB, while *yellow dashed line* corresponds to the lower molecular weight peak fraction (Peak 3). *C*, SEC-MALS profile of EspB_1–332_ obtained after two-step purification. *Blue trace* denotes refractive index, *black trace* indicates the molar mass, and *red trace* represents the molar mass fit. *D*, negative staining TEM visualization of the three distinct peak fractions marked in the SEC profile. From *left*, Peak 1 indicates the presence of multiple fused ring-like oligomers that range from dimers to higher order associations. *Bottom panel* denotes two representative 2D class averages (enlarged from Fig. S2*A*) that show joined rings in the multiples of two and three, respectively. Next, Peak 2 comprises the most homogeneous distribution of oligomeric EspB_1–332_. Representative 2D class averages (enlarged from Fig. S2*B*) show the top and side views of ring-like EspB_1–332_. To the *right* is provided a visualization of Peak 3 that comprises open chain form of EspB_1–332_. Magnified 2D class averages (enlarged from Fig. S2*C*) indicate the flexible nature of these chain-like EspB. Scale bars indicate 10 nm. *Cyan arrow* marks the area of bending of the protein. *White arrowheads* have been used to highlight the different oligomeric assemblies in the raw micrographs. Lane 1, Ni-NTA purified protein; Lane 2, SEC purified protein fractions pooled; M, marker; MALS, multi-angle light scattering; SEC, size-exclusion chromatography; TEM, transmission electron microscopy.

Previous reports demonstrated that 1 to 332 amino acids of EspB of Rv3881c interacts with phospholipids (27). Therefore, the coding sequence corresponding to amino acids 1 to 332 of Rv3881c was amplified from the genomic DNA of *M. tuberculosis* H37Rv and cloned into pET28a vector for heterologous overexpression in *Escherichia coli* BL21(DE3) cells (Fig. S1*A*). The clone length, as described previously (27), was particularly selected to study the interaction of EspB_1–332_ with PA and PS (Fig. S1*A*). Despite truncating most of the disordered residues reported for the C-terminal region of the protein, the amino acid sequence indicated a disorder content of nearly 43%, while the remaining predominantly adopted alpha helical structures (Fig. S1*B*). Assessment of the alpha fold secondary structure prediction of the construct showed that both termini of EspB_1–332_ contained disordered stretches, approximately ranging between Met1-Asp10 for the N-terminal and residues Ala269-Pro332 for the C-terminal (Fig. S1, *C* and *D*). Although high-resolution structural characterization could be limited by the presence of such large content of disordered and flexible residues, we proceeded to study EspB_1–332_, resembling secreted EspB, in the presence of phospholipids. At first, recombinant EspB_1–332_ was overexpressed and purified by Ni-NTA affinity chromatography (Fig. 1*A*). The recombinant protein was trypsin digested and was identified to be EspB through MALDI-TOF mass spectrometry (Fig. S1*E*). To further improve the homogeneity of purified protein, size-exclusion chromatography (SEC) was performed. The gel filtration elution profile was distributed in a majorly trimodal pattern with a small peak separating the two major peaks (Fig. 1*B*). This is in agreement with previous studies citing multiple oligomeric states of EspB (20, 21, 23). For better understanding the oligomeric distribution of EspB_1–332_, the SEC-purified fractions were pooled and analyzed with SEC coupled with multi-angle light scattering (MALS). We obtained four distinct peaks roughly corresponding to predominant molecular weights of 2.2 MDa, 480 kDa, 251 kDa, and 36 kDa, starting from the earliest eluting peak (Fig. 1*C*). Toward obtaining a visual representation of the nature of oligomers, we took the three peak fractions from SEC, namely peak 1 at 9 ml, peak 2 at 11.4 ml, and peak 3 at 13.8 ml to observe the particle distribution under room temperature (RT) negative staining transmission electron microscopy (NS-TEM). Our microscopy data revealed intriguing patterns of EspB_1–332_ protein distribution in solution. Peak 1 comprised multiples of ring-like heptamers that associate together in orders of 2, 3, 4, and higher to form the first eluting broad fraction observed in SEC (Figs. 1*D* and S2*A*). Peak 2 displayed the usual heptameric, homogeneously distributed EspB_1–332_ ring-like oligomers (Figs. 1*D* and S2*B*). From the 2D class averages, the ring-like top views and the vase-like side views were distinctly discernible. Finally, TEM inspection of peak 3 revealed unique filamentous organizations of EspB_1–332_ approximately ranging between 12 and 14 nm in length (Figs. 1*D* and S2*C*), possibly corresponding to monomeric form of EspB_1–332_.

### Single particle cryo-EM 3D reconstruction of purified EspB_1–332_

**Figure 2:**
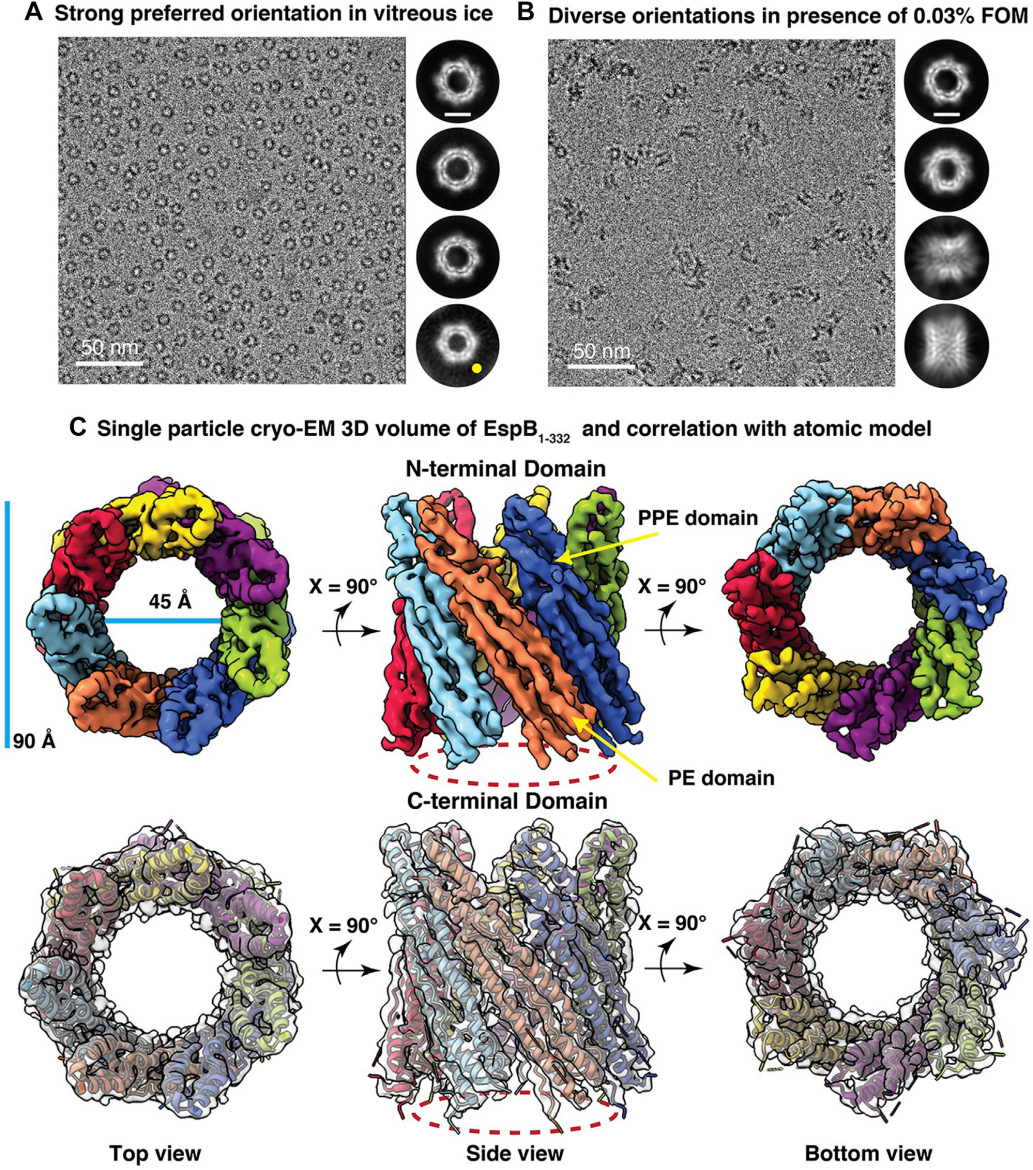
**Single-particle cryo-EM structure determination of secreted EspB.***A*, cryo-EM raw micrograph showing a biased orientation distribution of EspB_1–332_ in amorphous ice. Adjacent 2D class averages show only top views of the protein. A minor subset of particles (marked *yellow*) illustrates the presence of hexameric EspB_1–332_ in a predominantly heptameric population. Scale bar denotes 5 nm. *B*, cryo-EM raw micrograph represents the effect of ∼0.03% fluorinated octyl maltoside on the orientation distribution in amorphous ice. Adjacent 2D class averages show the appearance of side views and tilted along with the previously observed top views. Scale bar denotes 5 nm. *C*, 3D density map of EspB_1–332_ resolved at 4.5 Å reveals strong agreement with the atomic model derived from full-length EspB_1–460_ (PDB ID 6XZC). *Upper panel* shows the cryo-EM structure where each monomer is colored differently to highlight the heptameric assembly of the N-terminal domain. The density corresponding to the C-terminal which could not be observed in our map has been traced by *dashed red* boundary. Transparent rendition of the cryo-EM structure in the *bottom panel* shows the quality of fitting of the secondary structures into the density map.

To evaluate whether the stacking of multiple EspB_1–332_ heptamers could confer upon ESX-1 system’s ability to cause virulence in a contact-dependent form, we aimed at performing single particle cryo-EM 3D reconstruction of the conjoined EspB_1–332_ rings. For this, we recorded multiframe cryo-EM movies using a 200 kV Talos Arctica equipped with direct electron detector. However, we consistently observed that the number of joined rings significantly reduced, and most of the protein was distributed as free single ring-like particles on the cryo-EM grids (Fig. S3). These cryo-EM data suggest that EspB multimers (peak 1) were extremely unstable at cryogenic environment and these multimeric interactions were extremely weak, which might easily dissociate at near-physiological cryogenic conditions. Therefore, we did not proceed with extensive data collection because it is possible that even though a significant proportion of EspB_1–332_ exists as fused rings in solution, the stability of these oligomers may be reduced under cryogenic condition and vitreous ice. Next, we sought to obtain structural insights into the discrete heptamers of C-terminal–processed EspB_1–332_. Much like full-length EspB heptamers, EspB_1–332_ also showed strong preferred orientation on the cryo-EM grids presenting only top views (Fig. 2*A*). This was a stark deviation from the homogeneous orientations observed in our negative staining images (Fig. 1*D*). However, it was interesting to note that the reference-free 2D class averages pointed out a minor subset of hexamers that amounted to nearly 1% of the total particles (Fig. 2*A*). Through cryo-EM, we were thus able to identify additional oligomeric stoichiometry among the isolated ring-like EspB_1–332_ population, highlighting the preference of EspB_1–332_ to exist in myriad associations and assemblies. Since orientation bias poses a severe bottleneck in high-resolution 3D structure determination, we implemented 0.03% fluorinated octyl maltoside (FOM) to alleviate particle adsorption at the air-water interface. Consequently, we were able to obtain the multiple orientations of EspB_1–332_ in the presence of surfactant. The uniform distribution of particles across the cryo-EM grid encouraged us to perform 3D reconstruction of EspB_1–332_. After multiple rounds of particle curation, we were able to resolve the structure of EspB_1–332_ at a global resolution of 4.5 Å (Fig. S4). From our cryo-EM density map, we could distinctly observe the PE and PPE domains harbored in the N-terminal of each EspB_1–332_ monomer. EspB_1–332_ heptamers had an outer diameter of nearly 90 Å and an inner diameter of nearly 45 Å. Thus, truncation of the greater part of the C-terminal domain did not affect the dimensions or N-terminal domain architecture of EspB_1–332_ (Fig. 2*C*). The density for the remaining C-terminal region, which remains unstructured, was undetected in our EspB_1–332_ map thereby reiterating the extent of disorder present. Overall, the current structure shares strong consensus with existing high-resolution characterizations of EspB and therefore it can be considered as a suitable control to study EspB_1–332_ in the context of lipid, which we have discussed in the following sections.

### C-terminal–processed EspB_1–332_ binds membranes composed of PA

**Figure 3:**
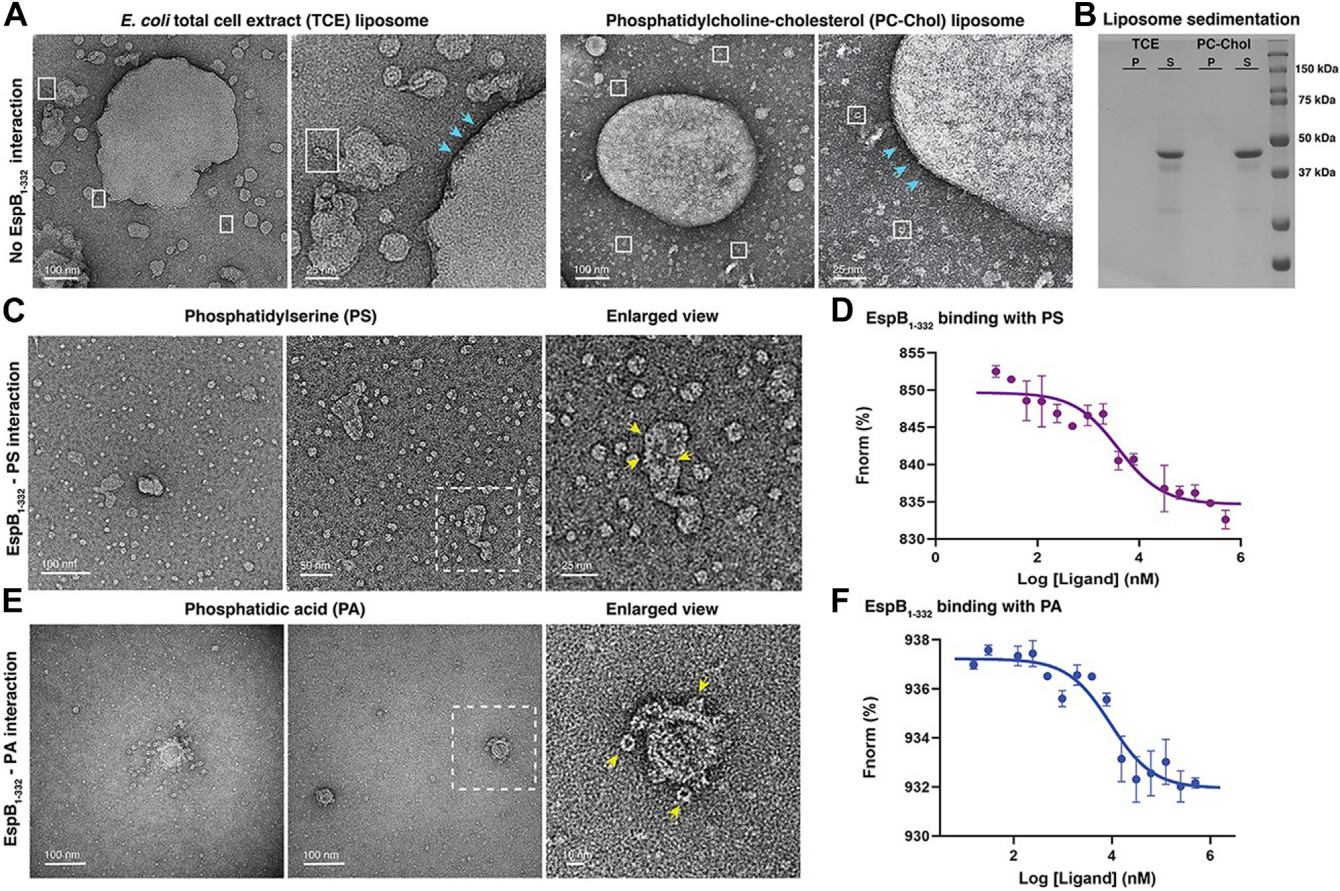
**Lipid-binding affinity of C-terminal–processed EspB**_**1–332**_**.***A*, *left*, a representative negative staining raw micrograph of EspB_1–332_ incubated with liposomes made of *Escherichia coli* total cell extract and enlarged view of the *top left corner*. *White boxes* are used to denote the protein and *cyan arrows* show the boundary of the TCE membrane devoid of any EspB_1–332_ particles. *Right*, a representative negative staining raw micrograph of EspB_1–332_ incubated with liposomes made of phosphatidylcholine and cholesterol in equimolar ratio and enlarged view of the bottom left corner. *White boxes* are used to denote the protein and *cyan arrows* show the boundary of the PC-Chol membrane devoid of any EspB_1–332_ particles. *B*, 12% SDS-PAGE following TCE and PC-Chol liposome sedimentation assay. Pellet fraction is denoted by P and supernatant is denoted by S. *C*, negative staining raw micrograph of EspB_1–332_ incubated with phospholipid phosphatidylserine. *Yellow arrows* indicate the EspB_1–332_ oligomers that appear to bind plausible lipid architecture. *D*, fluorescence-based binding assay shows binding affinity of EspB_1–332_ with PS molecules, detected in MST mode. *E*, negative staining raw micrograph of EspB_1–332_ incubated with phospholipid phosphatidic acid. *Yellow arrows* indicate the EspB_1–332_ oligomers that appear to bind plausible lipid architecture. *F*, fluorescence-based binding assay shows binding affinity of EspB_1–332_ with PA molecules, detected in MST mode. Data presented here are mean ± SD from two independent sets of protein purification. MST, microscale thermophoresis; PA, phosphatidic acid; PC-Chol, phosphatidylcholine-cholesterol; PS, phosphatidylserine; TCE, total cell extract.

Chen *et al.*, 2013 (27) had proposed that the mature form of EspB, comprising residues 1 to 332, had a preference to bind lipids like PA and PS, unlike the full-length EspB_1–460_. However, the way in which secreted EspB can bind PA or PS remains an unanswered question. The presence of a localized stretch of positively charged amino acids in the interior of EspB had invoked a hypothesis that anionic single phospholipids such as PA and PS may be transported through the channel-like N-terminal domain (22). To delve further into the mechanism of phospholipid interaction of C-terminal–processed EspB_1–332_, we probed binding with phospholipids, PA and PS, and liposome preparations. At first, we visualized membrane interaction of EspB_1–332_ in the context of *E. coli* total cell extract (TCE) liposomes and phosphatidylcholine-cholesterol (PC-Chol) liposomes. For this, heptameric EspB_1–332_ incubated with TCE and PC-Chol liposomes were briefly spun down. Analysis of the resultant supernatant and pellet samples *via* NS-TEM revealed that the greater proportion of EspB_1–332_ remained in the supernatant, while the pellet comprising the liposomes were unassociated with EspB_1–332_ (Figs. 3*A* and S5). The protein particles that were visible in the pellet fractions were present in the background remaining distinctly excluded from the boundaries of both the TCE liposomes and PC-Chol liposomes. *E. coli* cell membranes are composed of anionic phospholipids, like phosphatidylglycerol and cardiolipin (CL), therefore the lack of EspB_1–332_ binding to such liposomes concurs with past findings (27). Similarly, PC-Chol liposomes also provide a control to confirm that EspB_1–332_ does not have affinity for PC or Chol. To further verify the observations, we performed liposome cosedimentation assay followed by SDS-PAGE. Neither TCE nor PC-Chol liposomes showed colocalization of EspB_1–332_ in the pellet fraction comprising the liposomes (Fig. 3*B*). This strongly support our previous observations that EspB_1–332_ is unable to interact with TCE and PC-Chol. Next, we incubated freshly purified EspB_1–332_ heptamers with single phospholipids PA and PS. In the case of PS, we observed that the protein heptamers were homogeneously distributed throughout the micrographs and intermingled with heterogeneously sized clusters of what could be PS molecules (Fig. 3*C*). To confirm the lipid–protein interactions, we prepared two-fold serial dilutions of PS solution and performed MST assay with fluorescently tagged heptameric EspB_1–332_. Our MST results indicated moderate binding affinity towards PS molecules (Fig. 3*D*). In contrast, for PA-treated EspB_1–332_, the NS-TEM micrographs showed unique pattern in which the heptamers were dispersed on the TEM grid. Unlike PS molecules, PA appeared to form small, ordered vesicles and interestingly, the protein particles were prominently localized near or around these lipid structures (Fig. 3*E*). Thus, visual inspection of heptameric EspB_1–332_ incubated with PA laid preliminary evidence of a lipid-binding disposition of this ESX-1–secreted substrate. This was additionally supported by MST (Fig. 3*F*) and gel-based–binding assay (Fig. S6).

### PA, PS binding facilitates conversion of filament-like EspB_1–332_ monomers to ring-shaped oligomeric EspB_1–332_

**Figure 4:**
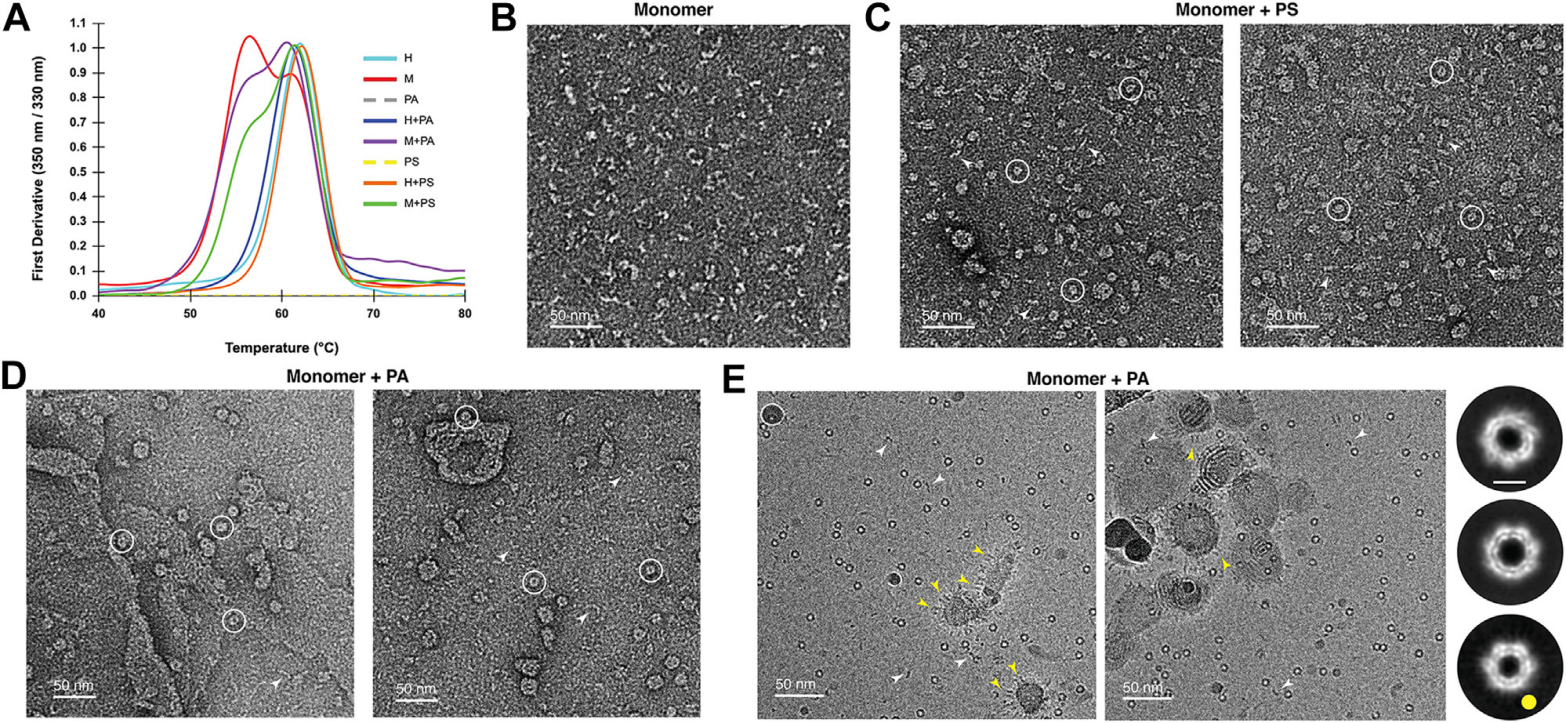
**PA and PS binding initiates conversion of EspB**_**1–332**_**monomers to oligomers.***A*, thermal melt spectra of EspB_1–332_ monomers and heptamers in the presence and absence of PA and PS. *Cyan line* denotes EspB_1–332_ heptamer, *red line* denotes EspB_1–332_ monomer, *gray dashed line* denotes only PA, *navy blue line* denotes EspB_1–332_ heptamer with PA, *purple line* denotes EspB_1–332_ monomer with PA, *yellow dashed line* shows only PS, *green line* shows EspB_1–332_ heptamer with PS, and *green line* shows EspB_1–332_ monomer with PS. *B*, NS-TEM raw micrograph of monomeric fraction of EspB_1–332_. *C*, NS-TEM raw micrograph of EspB_1–332_ monomer with phosphatidylserine. Oligomers are shown within *white circles*, and monomers are highlighted with *white arrowheads*. *D*, NS-TEM raw micrograph of EspB_1–332_ monomer with phosphatidic acid. Oligomers are shown within *white circles*, and monomers are highlighted with *white arrowheads*. *E*, cryo-EM raw micrograph of EspB_1–332_ monomer with phosphatidic acid. *Yellow arrowheads* denote oligomeric side-views adhered to PA vesicles, and monomers are highlighted with *white arrowheads*. Adjacent cryo-EM 2D class averages show a predominance of heptameric oligomers over hexameric oligomers (marked *yellow*) upon PA incubation. Scale bar denotes 5 nm. NS-TEM, negative staining transmission electron microscopy; PA, phosphatidic acid; PS, phosphatidylserine.

As revealed by our initial SEC and NS-TEM data, larger proportion of purified EspB_1–332_ existed in the form of monomers. This was also in accordance with Gijsbers *et al.* (23), who showed that acidic or phagosomal pH was required to trigger the conversion of EspB monomers to heptamers, indicating that heptamers were most likely the functional form of EspB implicated in pathogenesis. However, the entire population of monomers were not reported to convert to heptameric oligomer, which indicates that lower pH may play a role to convert EspB monomer to oligomer, but it may not be the sole factor for oligomerization. The first report on PA and PS binding was also assessed using the heptameric fraction of EspB_1–332_ (27). The effect of lipid binding to the monomeric population however remains elusive to date. To address this, we sought to understand whether PA or PS binding could render additional stability to the EspB_1–332_ monomers. Upon performing thermal denaturation of EspB_1–332_ monomers, we observed two distinct peaks at Tm = 56.4 °C and 61.1 °C, signifying the presence of at least two unique populations occurring in different amounts (Figs. 4*A* and S7*D*). Surprisingly, for both PA and PS incubated samples, there appeared to be a decrease in the population at Tm ∼56 °C along with an increase in the species with Tm ∼61 °C (Figs. 4*A* and S7, *D*–*F*). Incidentally, the Tm of the increasing population aligned with that observed for heptameric EspB_1–332_, Tm = 62 °C. (Fig. S7*A*). When studied with TCE or PC lipids, this trend of decrease in population with Tm ∼56 °C with concomitant increase in the species with Tm ∼61 °C was not observed (Fig. S8). For heptameric EspB_1–332_, however, we could not observe significant change in the Tm value with or without lipid incubation, ΔTm with PA = −0.7 °C and ΔTm with PS = +0.1 °C (Figs. 4*A* and S7, *A*–*C*). Although PA or PS binding did not confer more stability to EspB_1–332_ heptamers, the impact of lipid incubation with the monomers was intriguing. To better understand this phenomenon, we performed NS-TEM to visualize the behavior of monomers in the presence of PA and PS. Remarkably, we were able to observe circularization of the linear EspB_1–332_ monomers (Fig. 4*B*) in the presence of anionic phospholipids PA and PS (Fig. 4, *C* and *D*). Our micrographs revealed the presence of both linear and ring-like oligomers of EspB_1–332_ indicating the conversion of the monomers to probable heptamers. Our previous TEM data indicated better localization of EspB_1–332_ heptamers with PA, therefore, to obtain high-resolution information about the newly converted oligomers, we performed cryo-EM of EspB_1–332_ monomers with PA. The cryo-EM raw micrographs showed distinct ring-like particles along with a few thread-like densities that could be the unconverted monomers. Consistent with our TEM data (Fig. 3*E*), we could identify multiple side-views of the EspB_1–332_ oligomers surrounding the PA vesicles (Fig. 4*E*). Upon performing reference-free 2D class averaging, we found that the majority of the oligomers were heptameric, while nearly 8% of the remaining population showed the presence of hexameric EspB_1–332_. This noteworthy increase in the hexameric population from less than 1% (Fig. 2*A*) to nearly 8% (Fig. 4*E*) also could imply the transition of monomers to heptamers. These results indicated that at neutral pH too, it was most likely for the heptameric ring-like assemblies of EspB_1–332_ to be the functional form in the presence of proper lipid environment. Taken together, the tendency of EspB_1–332_ to associate with PA assemblies and the preferential conversion of EspB_1–332_ monomers to ring-like heptamers encouraged us to further explore EspB_1–332_ heptamers with PA.

### Single particle cryo-EM 3D reconstruction of heptameric EspB_1–332_ with PA

**Figure 5:**
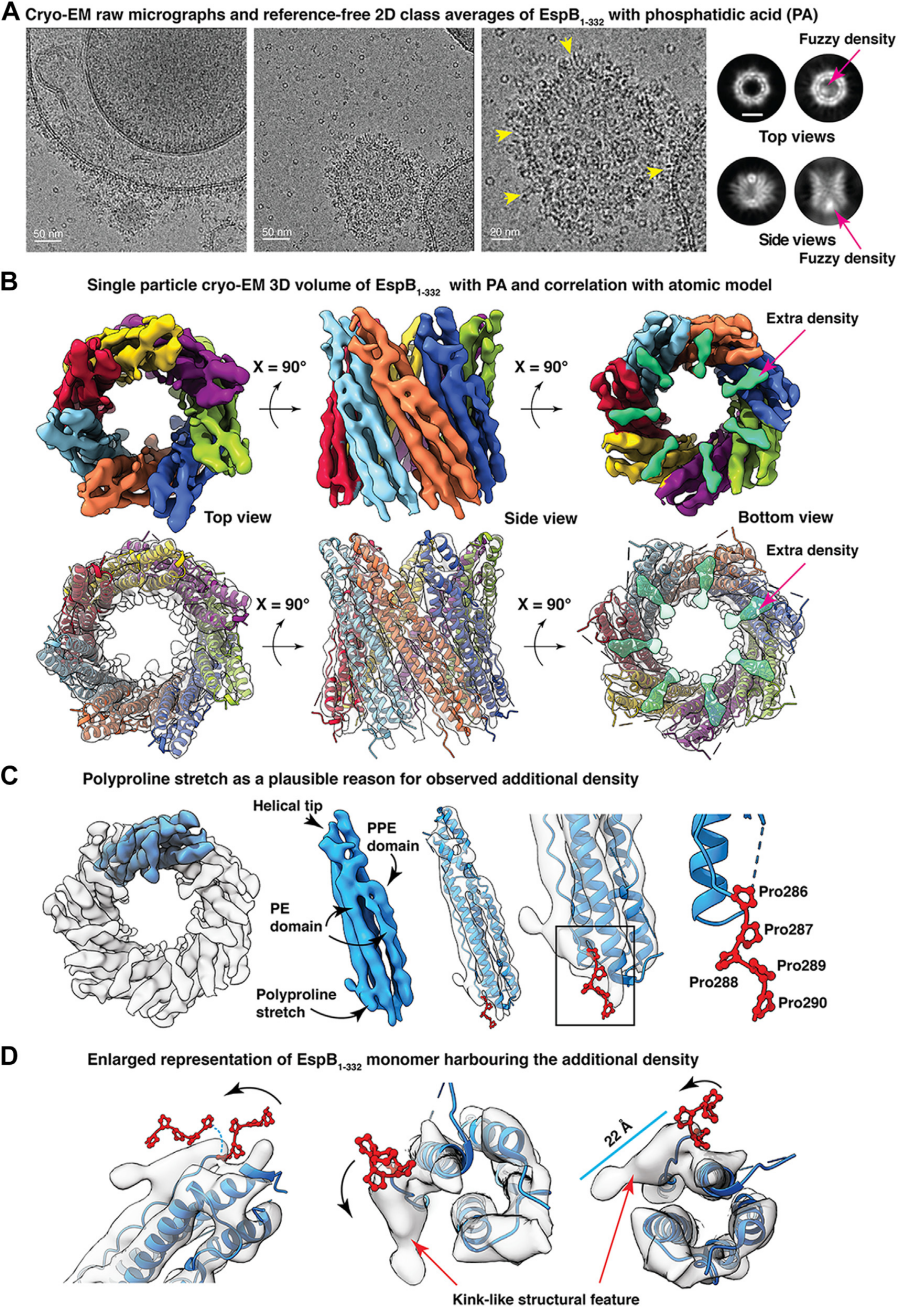
**Secreted EspB adheres to membrane bilayer formed by phosphatidic acid.***A*, cryo-EM raw micrographs (*left-hand* and *center panels*) from two different fields show preferential attachment of EspB_1–332_ heptamers on the PA vesicle surface. For clarity, an enlarged view of the *central* panel is presented in the third image panel (*right*), and *yellow arrowheads* have been used to pinpoint the side views of the protein attached on the membrane surface. Reference-free 2D class averages show different orientations of the heptamers. Particular top and side views have been highlighted because of the appearance of a fuzzy density at the *bottom* of the channel. Scale bar denotes 5 nm. *B*, 3D density map of EspB_1–332_ resolved at 6.6 Å indicates coherence with the atomic model derived from full-length EspB_1–460_ (PDB ID 6XZC). *Upper panel* shows the cryo-EM structure where each monomer is colored differently to highlight the heptameric assembly of the N-terminal domain. The additional density visible in the *bottom* view of the map has been colored *green*. Transparent rendition of the cryo-EM structure in the *bottom panel* shows the absence of modeled coordinates that could fit into the bottom density marked *transparent green*. *C*, transparent rendition of the electron density map where a single monomer has been colored *blue*. Monomer derived from the cryo-EM map has been used to show the prominent domains – PE domain, helical tip, PPE domain, and the polyproline stretch. Transparent rendition of the monomer fitted with a single EspB chain from PDB 6XZC illustrates the flexible string of multiple prolines. *D*, different orientations of the magnified view of the additional density linked to the helical N-terminal domain. *Black curved arrows* have been used to show the flexibility of the polyproline stretch. *Blue dashed line* in the first panel shows a hypothetical trajectory of motion of the prolines to continue into the ∼22 Å–long extra density which could correspond to the C-terminal domain. PA, phosphatidic acid.

**Figure 6:**
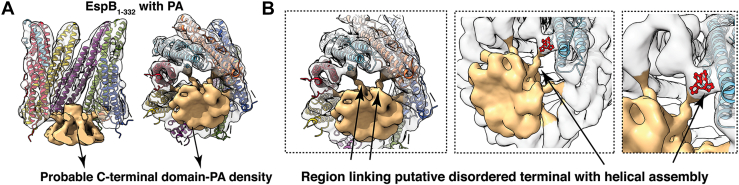
**Stabilization of the disordered C-terminal domain in the presence of lipid bilayer and plausible lipid-binding sites within the EspB**_**1–332**_**core.***A*, representation of the additional density (marked *golden*) in the PA-EspB_1–332_ cryo-EM map fitted with PDB 6XZC. *B*, tilted *bottom* view of the PA-EspB_1–332_ structure shows that the low-resolution bottom extra density is connected to the well-resolved N-terminal domain. Succeeding representations indicate that the proline-enriched loop may serve as the link joining the putative C-terminal domain (here, *golden* density) with the helical N-terminal assembly of EspB_1–332_. PA, phosphatidic acid.

With the objective of exploring secreted form of EspB and PA in a near-native environment, we collected a total of 2601 multiframe cryo-EM movies. The tendency of PA to associate into vesicles remained unaltered under cryogenic conditions and remarkably, we observed the formation of large unilamellar vesicles like structures of various sizes, uniformly occurring throughout vitreous ice (Figs. 5*A* and S9). Consistent with our NS-TEM impression, almost all of the EspB_1–332_ heptamers flocked to the PA vesicles leaving very few isolated heptamers in buffer (Figs. 5*A* and S9). The vesicles were densely decorated with top views of EspB_1–332_ as well as side views present along the bilayer regions formed by PA. It is also noteworthy to mention that the presence of PA attenuated preferred orientation of EspB_1–332_ alone in amorphous ice. This fortuitous potential of C-terminal–processed EspB_1–332_ to be able to specifically adhere to bilayer structures made of PA urged us to examine the structural changes, if any, upon membrane binding. Toward this goal, we curated an initial set of 751,763 membrane-bound EspB_1–332_ particles. Reference-free 2D class averages showed multiple orientations of the protein—top, tilted, and side views (Fig. 5*A*). However, we were unable to visualize the distinct bilayer of PA vesicles in the class averages corresponding to the side view of EspB_1–332_ (Fig. 5*A*). Unlike transporters or various pore-forming toxins (29, 30), which embed firmly in between the two leaflets of membrane-bilayer, it is possible that EspB_1–332_, despite having spontaneous binding affinity to PA bilayer, may adhere superficially to the lipid surface. This could potentially lead to averaging out of the signal corresponding to lipid density during the process of particle alignment. Nevertheless, we went ahead with cryo-EM 3D reconstruction of EspB_1–332_ with PA and were able to obtain the structure at a global resolution of 6.6 Å where the PE and PPE domains in the N-terminal domain were well resolved (Figs. 5*B* and S10). Fitting the atomic model of EspB (PDB ID: 6XZC) (22) into our cryo-EM map hinted at strong correlation between the two structures, indicating that PA binding did not cause any major movement or conformational changes in secondary structures pertaining to the N-terminal region of EspB_1–332_ (Fig. 5*B*). Extending beyond the PPE domain, we observed additional densities in the C-terminal region for which the atomic coordinates have not been modeled (Fig. 5*B*). Further analyzing the connectivity of this ∼22 Å long extra density indicated that it may be a continuation of the polyproline stretch that links the N-terminal folded region with the C-terminal unstructured part. (Fig. 5, *C* and *D*). Successively present prolines may potentially induce flexible motion in the loop, allowing it to bend resembling the additional density, thereby leading to the C-terminal domain (Fig. 5, *C* and *D*). However, owing to the high flexibility of disordered residues, application of a uniform B-factor to sharpen the cryo-EM map compromised the connectivity of the dynamic C-terminal region at the given threshold of volume. Particularly in the PA-treated dataset, we consistently observed a firm density localized at the bottom of the heptameric ring. This density was present in all the 3D maps including the unfiltered half-maps of the final structure (Fig. S10). Thus, to improve the interpretability of the structure, we used DeepEMhancer (31) to sharpen the unfiltered half-maps of both the control EspB_1–332_ map as well as to the PA-treated structure. On viewing the resultant EspB_1–332_ maps at different levels of volume, we observed that the control EspB_1–332_ structure was devoid of any bottom density even at the lowest threshold of density. On the other hand, the appearance of an extra density at the bottom of the PA-EspB_1–332_ map was maintained at all thresholds, getting more pronounced with every step of boosting the volume (Fig. S11). Consistent with our previous observation, the pronounced bottom density was firmly linked to the N-terminal region (Fig. 6, *A* and *B*). Curiously, this extra density which was exclusive to the PA-EspB_1–332_ map was located at a position equivalent to where Piton *et al.* (22), had marked the low-resolution C-terminal domain density in their full-length EspB structure (Figs. 6, *A* and *B* and S11).

### Characterization of EspB_1–332_ in the context of model membranes comprising PA and PS

**Figure 7:**
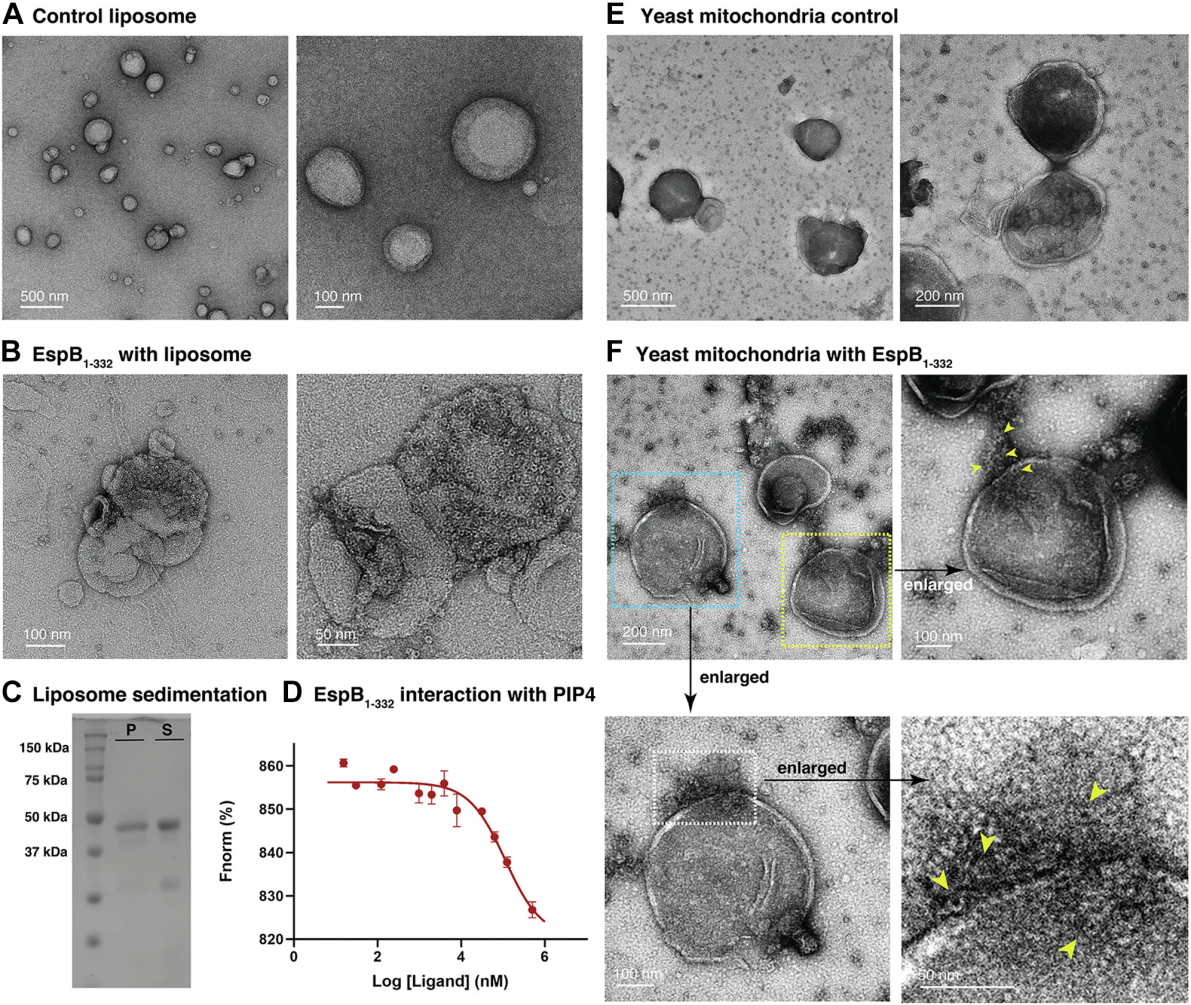
**Biophysical characterization of secreted isoform of EspB**_**1**–**332**_**in the presence of model membranes.***A*, negative staining raw micrographs show liposomes made of PC, phosphatidylethanolamine, PIP4, PA, PS, and CL. *B*, negative staining raw micrograph shows liposomes made of PC, phosphatidylethanolamine, PIP4, PA, PS, and CL covered by ring-like EspB_1–332_. *C*, 12% SDS-PAGE following model liposome sedimentation assay. Pellet fraction is denoted by P and supernatant is denoted by S. *D*, fluorescence-based binding assay shows binding affinity of EspB_1–332_ with PIP4 molecules, detected in MST mode. *E*, control *Saccharomyces cerevisiae* mitochondria observed under NS-TEM. *F*, NS-TEM raw micrograph showing three isolated mitochondria post incubation with EspB_1–332_. Two mitochondria (marked with *cyan* and *yellow box*) have been enlarged, in the *top right* and *bottom left panels*, where distinct islands of EspB_1–332_ binding can be observed (marked with *yellow arrowheads*). *Bottom right panel* is an enlarged representation of the *bottom left panel*. Data presented here are mean ± SD from two independent sets of protein purification. CL, cardiolipin; MST, microscale thermophoresis; NS-TEM, negative staining transmission electron microscopy; PA, phosphatidic acid; PIP4, phosphatidylinositol-4-phosphate; PS, phosphatidylserine.

Ensuing our observation that EspB_1–332_ possesses membrane bilayer–binding property, we sought to study the affinity of EspB_1–332_ towards membranes posing a closer resemblance to host cell environment, in terms of membrane curvature and composition. It is interesting to note that most pathogens, including *M. tuberculosis*, mediate pathogenesis by causing host mitochondrial damage where mitochondrial fission and fusion is severely affected (32, 33, 34). Moreover, PA and PS are also known markers of apoptosis. Of particular importance here is PA which regulates intracellular Ca^2+^ concentration (35), one of the primary signals to induce apoptosis (36). Thus, we hypothesized that role of EspB_1–332_ as an ESX-1 substrate may be to elicit host mitochondrial damage by binding PA and PS present in the outer cell membrane of mitochondria. To test this hypothesis, we prepared liposomes which roughly satisfy the phospholipid proportions of mitochondrial outer leaflet (37). First, we visualized the model membranes using NS-TEM which showed intact liposomes of diameter of ∼100 nm and above (Fig. 7*A*). Having checked the morphology of the control liposomes, we incubated the same with freshly purified EspB_1–332_ heptamers. Surprisingly, we found a biased distribution of the protein particles such that the liposomes were crowded with ring-like EspB_1–332_ molecules with few isolated proteins in the background (Fig. 7*B*). This tendency was maintained all over the NS-TEM grid (Fig. S12*A*). However, it is noteworthy to mention that despite having only 3% of PA and PS combined in the lipid membrane, we were able to obtain very homogenous attachment of EspB_1–332_ over the liposome surface. Further confirmation of membrane interaction was obtained through liposome cosedimentation assay, which indicated colocalization of a fraction of EspB_1–332_ with the model membrane (Fig. 7*C*). Thus, even when PC and phosphatidylethanolamine were the main components determining the membrane curvature, the large unilamellar vesicle–binding ability was not impeded. To investigate the role of each lipid component in driving the interaction of EspB_1–332_ heptamers with the model liposome, we performed binding assay of fluorescently labeled heptameric EspB_1–332_ with individual lipids. We observed binding affinity with PIP4, while the other components of the model membrane, phosphatidylethanolamine, PC, and CL, showed no affinity, thereby indicating that binding of EspB_1–332_ with our model membrane was governed by PA and PS, along with PIP4 (Fig. S12*B*). Now, to determine whether this membrane-binding property of secreted EspB could cause rupture of model membrane, we attempted to estimate membrane permeabilization using carboxyfluorescein (CF) release assay. However, we could not record any leakage signal over multiple time frames of the experiment (Fig. S13). Through this, we were able to rule out the possibility of any membrane-damaging activity of EspB. However, EspB might damage actual mitochondrial membrane through some specific receptors or proteins, which are located in mitochondrial membrane. Moreover, we were unable to reach any particular conclusion of membrane damage using our artificial membranes. Finally, to validate our findings in a model-membrane system, we incubated EspB_1–332_ with yeast mitochondria which closely resembles human mitochondria in terms of cell membrane composition. Initially, NS-TEM showed the presence of intact yeast mitochondria where the membrane bilayer appeared continuous (Figs. 7*E* and S14*A*). In the presence of EspB_1–332_, our TEM data revealed distinct localizations of EspB_1–332_ heptamers over the mitochondrial cell membrane (Fig. 7*F*). A particularly interesting observation was the altered morphology of yeast mitochondria post treatment with EspB_1–332_ (Fig. S14). A significant number of mitochondria appeared to clump together, possibly fusing into large aggregates (Fig. S14*B*) while some appeared to be lying on a layer of leaked substances (Fig. S14*C*). Thus, through our NS-TEM data, we infer that EspB_1–332_ tends to bind yeast mitochondrial membrane and may even impact the intactness of the mitochondrial membrane. However, further cell-based assay is required to validate our TEM and cryo-EM based observations.

## Discussion

EspB, the largest PE/PPE family substrate secreted by the ESX-1 system of *M. tuberculosis*, is known to be a key player in mediating host cell pathogenicity. A wealth of structural information shows the architecture of the N-terminal region, PE and PPE domains comprising the YxxxD and WxG motifs respectively, a linker joining the PE/PPE domains, and a helical tip (20, 21, 22, 23). However, to date, the C-terminal domain remains unstructured. It was earlier thought that the long C-terminal (Pro280-Lys460) causes steric hindrance and prevents the monomers of EspB from oligomerizing within the *M. tuberculosis* cytosol, that is why heptameric EspB is undetected in the cell lysate (21). It has now been shown that EspK plays the role of a chaperone that interacts with the helical tip of EspB_1–460_ and prevents premature oligomerization of EspB_1–460_ in the cytosol until it is cleaved by MycP_1_ and secreted through the inner membrane complex (38). Once EspB_1–460_ is cleaved at Pro332 and A392 by the serine protease MycP_1_ (25, 39), mature form of EspB is secreted into the culture filtrate, where it exists predominantly as heptamers. Mutation of Pro332 or Ala392 was shown to be detrimental to the growth of *M. tuberculosis* or *Mycobacterium marinum*, suggesting an exclusive role of these amino acid residues in Mycobacterial survival (39).Findings by Chen *et al.* 2013 (27), also impressed upon the importance of the C-terminal domain by showing that EspB_1–332_ could selectively bind PA and PS, while the EspB_1–460_ with longer stretch of disordered residues did not show lipid-binding property. In our current study, we show that EspB_1–332_ binds PA and PS in the context of membrane bilayers. Present experiments with the monomeric fraction of EspB_1–332_ and PA or PS lipid preparation reveal lipid-dependent oligomerization of the virulence factor. EspB heptamers have long been studied as the functional assembly of the ESX-1–secreted substrate. Taking our observations into account, it may be possible that maintaining a large proportion of linear monomers at physiological pH may be a checkpoint in regulating off-target virulence. The equilibrium of EspB species which is largely shifted towards the monomeric state at neutral pH may be reversed towards the oligomeric state once EspB comes in contact with PA and/or PS molecules at the site of action, which may be host organelle membrane. This pH-independent lipid-induced monomer to oligomer conversion is an important phenomenon, which could impact the virulence property of EspB at physiological pH. Our cryo-EM structure determination showed that there was an intriguing difference in the C-terminal domain of the EspB_1–332_ control structure and the map obtained when EspB_1–332_ was treated with PA. The control protein sample resembled a hollow ring-like structure comparable to all the previously resolved structures of EspB. However, in the presence of PA, EspB_1–332_ showed a firm density at the location of the disordered C-terminal domain (Figs. 6, *A* and *B* and S11) (22), although the C-terminal domain was completely unresolved. The secondary structures that make up the ‘novel’ density in our map are not evident, but the overall architecture is reminiscent of the unexplained density that was observed by Solomonson *et al.*, 2015 (20) and proposed to be the C-terminal–disordered residues (Fig. 6*A*). Inherent disorder in the protein in association with structural rearrangements of the C-terminal residues may help explain why the resolution of our structure could not be improved further. Additionally, we did not implement mask during refinement of EspB_1–332_ to observe the presence of any extra density associated with PA-EspB_1–332_. This also affects our overall resolution. Despite the possibility that masked refinement may improve the global resolution, application of a mask around PA-EspB_1–332_ would remove the disordered region and any associated lipid density from the map. This in turn would hamper our target to observe lipid–protein interaction. Close inspection of the PA-EspB_1–332_ structure revealed that this ancillary density was a continuation of the polyproline stretch known to connect the N-terminal helical tip with the low-complexity C-terminal region (Figs. 5, *C* and *D* and 6, *A* and *B*). Taken together, we infer that this extra density present exclusively in the PA-EspB_1–332_ map could be the disordered C-terminal domain of secreted EspB. This phenomenon of partial structural rearrangements upon membrane interaction appears analogous to how the unstructured N-terminus of alpha synuclein (αSn) adopts a helical secondary structure upon binding negatively charged lipid bilayers (40, 41, 42). Similarly, it was shown for ADP-ribosylation factor GTPase activating protein 1 (ArfGAP1) that at low concentration, disorder to order transition of nearly 40 amino acid residues facilitated binding to target membrane (41, 43). Strikingly, the C-terminal of EspB_1–332_ is rich in disorder promoting residues such as alanine, glutamate, glutamine, glycine, lysine, proline, and serine (44) (Fig. 6, *B* and *C*). Therefore, it is tempting to consider that interaction with lipid brings order in the otherwise flexible C-terminal domain, that is why we were able to observe this region only in the PA dataset and not in the only EspB_1–332_ dataset.

**Figure 8:**
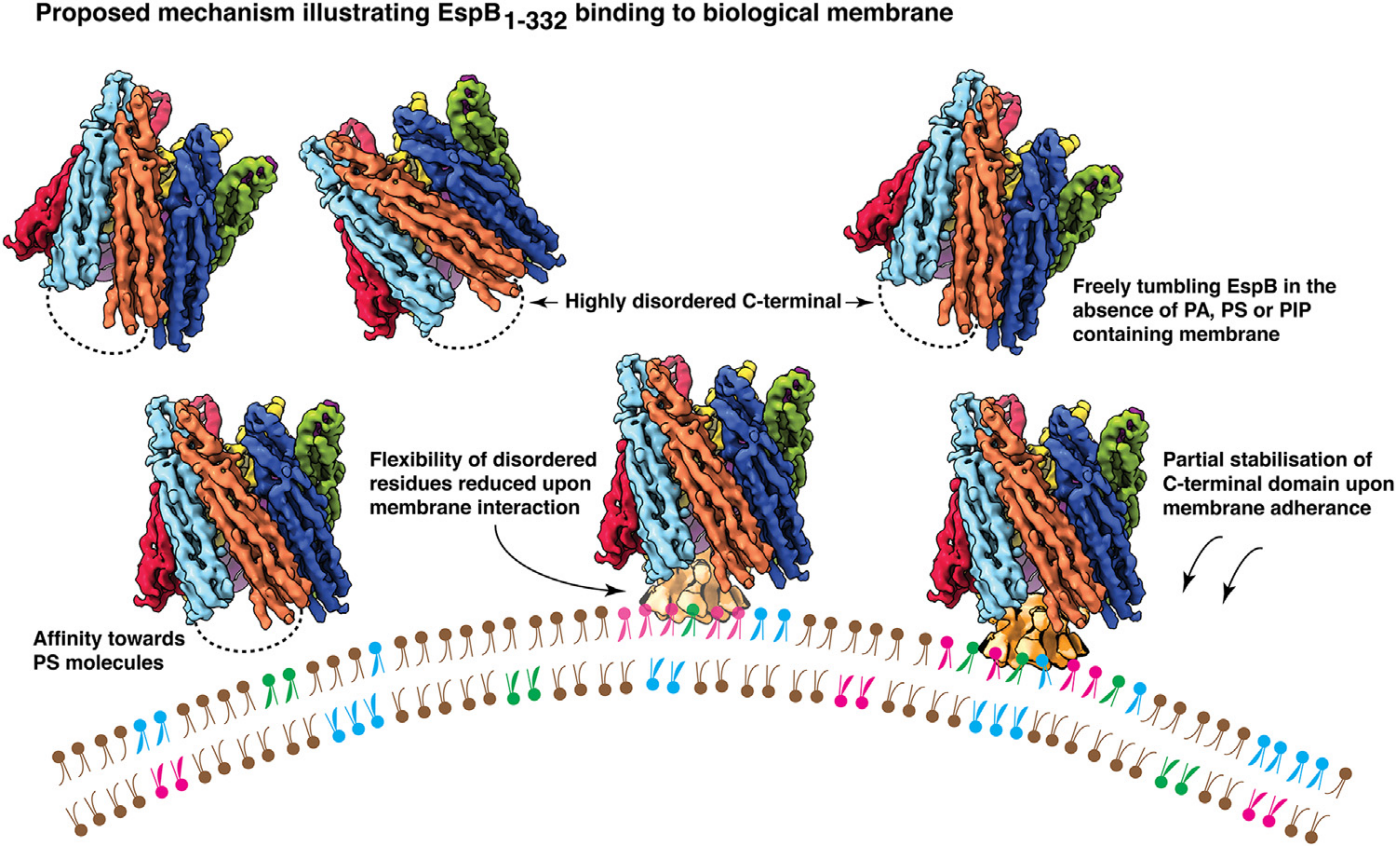
**Proposed mechanism of EspB**_**1–332**_**binding to biological membrane.** Schematic diagram illustrating a hypothetical basis for MycP_1_-processed EspB and host-membrane interaction. In brief, PA, PS, and/or PIP4 binding facilitates disorder to order transition in the low-complexity C-terminal domain. In figure, *pink color* has been used to denote PA molecules, *green color* has been used to denote PS molecules, and *blue color* has been used to denote PIP4 molecules. PA, phosphatidic acid; PIP4, phosphatidylinositol-4-phosphate; PS, phosphatidylserine.

Importantly, inspection of EspB with model membrane helped us to identify PIP4 as an additional target of EspB. PIPs are widely known as important signaling molecules and are implicated in mitochondrial fragmentation (45) and phagosomal maturation (46). Based on these results, we propose a hypothetical model by which MycP_1_-processed EspB may essay its role as pathogenic substrate of Mycobacterial T7SS system (Fig. 8). In solution, EspB oligomers freely tumble and are found in multiple orientations. However, the disordered C-terminal domain cannot be observed in such an environment owing to the dynamic nature of the amino acid residues. Moreover, as the heptameric ESX-1 substrate approaches host cell membrane, possibly mitochondrial outer cell membrane, PA, PS, and PIP4 affinity ushers EspB to associate with the host membrane. The interaction reduces the degree of freedom of the disordered C-terminal domain and stabilizes the residues thereby making the C-terminal domain visible post membrane binding. Thus, we speculate a folding-upon-binding mechanism of EspB-mediated cytotoxicity. Further experiments are required to accurately decipher if our hypothesis indeed holds true. However, it is noteworthy to consider that even though MycP_1_ cleaves most of the disordered domain, a sizeable stretch of over 50 disordered amino acid residues is still retained in the secreted isoform of EspB. Although the function of this low-complexity region has not yet been determined, our results combined with past findings indicate that the information to bind membrane is harbored in the C-terminal domain. This may help explain why Korotkova *et al.* (21), who resolved the crystal structure of EspB_7–278_ in presence of PS, could not obtain the density for PS. Nevertheless, the specificity of C-terminal domain toward lipid binding can be confirmed by truncating the disordered residues, which we intend to investigate extensively in the future. On the other hand, EspB may also have affinity for PI and other PIPs, as recently indicated (47), thereby playing a role in the prevention of phagosome maturation (26).

In summary, we have shown that EspB in a postsecretion heptameric state binds host membrane *via* interaction with PA, PS, and PIP4. PA and PS were also found to play a crucial role in triggering the oligomerization of EspB monomers at neutral pH. Cryo-EM structure characterization of heptameric EspB with PA revealed partial stabilization of the unstructured C-terminal domain upon PA interaction. Our cryo-EM data also suggests that EspB adheres to PA vesicles, plausibly by interaction with the lipid head groups, and does not embed firmly within the membrane environment. Liposome sedimentation assay and NS-TEM performed with model membrane and yeast mitochondria indicate affinity of EspB to bind to the mitochondrial outer membrane. Although we ruled out the possibility that EspB is a membranolytic virulence factor, it is possible that EspB elicits pathogenesis by binding lipids such as PA, PS, and inositol phosphates, which are important for cell signaling. Thus, host membrane–binding property poses EspB as an important target for developing alternative therapeutic measures against *M. tuberculosis*. Therapeutic compounds targeted against EspB–membrane interaction may show prospect in attenuating the survival of tubercle bacilli within host macrophages.

## Experimental procedures

### Cloning, expression, and purification of EspB_1–332_

The genomic DNA of *M. tuberculosis* was a kind gift from Prof. Amit Singh, Indian Institute of Science, Bangalore. Nucleotide sequence corresponding to residues 1 to 332 of Rv3881c was amplified using Phusion polymerase (NEB) and cloned into pET28a between NdeI and HindIII restriction sites. The clone was confirmed by sequencing and transformed into *E. coli* BL21(DE3) cells for recombinant protein overexpression.

For recombinant protein expression, the *E. coli* BL21(DE3) cells were grown in LB at 37 °C to A_600_ 0.6 and induced for 12 h with 0.5 mM IPTG at 16 °C. Cells were harvested by centrifugation and resuspended in buffer containing 20 mM Tris–HCl pH 8.0, 10 mM Imidazole, and 300 mM NaCl. The resuspended cells were lysed by sonication followed by centrifugation at 4 °C, 13,000 rpm for 1 h. Recombinant proteins were purified by Ni-NTA affinity chromatography. The cell lysate corresponding to each protein was loaded onto Ni-NTA column pre-equilibrated with the lysis buffer (20 mM Tris–HCl pH 8.0, 10 mM Imidazole, and 300 mM NaCl). The columns were initially washed with the lysis buffer followed by sequential washes containing 20 mM, 40 mM, and 80 mM Imidazole. Protein was eluted in a buffer containing 20 mM Tris pH 8.0, 300 mM NaCl, and 300 mM Imidazole. During elution, the elute was checked for the presence of protein using Bradford reagent. The protein was analyzed for purity in 12% SDS-PAGE gel. This was followed by SEC using 24 ml Superdex 200 Increase 10/300 GL column on an AKTA-FPLC system (GE Healthcare). Analytical gel filtration was performed in 20 mM Tris pH 7.5 and 150 mM NaCl at the flow rate of 0.5 ml/min.

### SEC coupled with MALS of purified EspB_1–332_

The fractions obtained from SEC were pooled and concentrated to ∼0.5 mg/ml. SEC–MALS was performed using 20 mM Tris pH 7.5 and 150 mM NaCl buffer–equilibrated analytical Superdex 200 Increase 10/300 GL gel filtration column (GE Healthcare) on a Shimadzu HPLC. Protein peaks resolved after size exclusion were subjected to in-line refractive index (Waters Corp) and MALS (mini DAWN TREOS, Wyatt Technology Corp) detection to estimate the molar mass. The data acquired from UV, MALS, and refractive index were analyzed using ASTRA 6.1 software (https://www.wyatt.com/products/software/astra.html; Wyatt Technology).

### Preparation of lipids and liposome sample

#### PA and PS preparation for microscopy and binding study and PIP4 for binding study

Powder form of 3-sn-PA (Sigma Aldrich), Brain PS (Avanti Polar Lipids), Brain PI-(4)-P (Avanti Polar Lipids) was dissolved in chloroform in a clean glass vial and evaporated in a desiccator overnight at RT. Thin film of dried lipid obtained after drying was dissolved in 20 mM Tris pH 7.5 and 150 mM NaCl and stored in −20 °C for future use.

### Liposome preparation

#### TCE liposome

Powder form of *E. coli* TCE (Avanti Polar Lipids) was dissolved in 20 mM Tris pH 7.5 and 150 mM NaCl and incubated at 55 °C for 30 min. This colloidal heated solution was subjected to extrusion (Mini Extruder, Avanti Polar Lipids) using 200 nm pore-sized polycarbonate membranes. Using the Avanti polar extruder apparatus, the colloidal solution was passed through the polycarbonate membranes for a total of eight rounds at 55 °C. The resultant clearer solution containing unilamellar vesicles were used for downstream experiments, and the remaining was stored at 4 °C for not more than 3 days.

#### PC-Chol liposome

Powder form of Egg-PC (Sigma Aldrich) and cholesterol (Sigma Aldrich) was dissolved in chloroform. Next, 250 μM chloroform–solubilized PC was mixed with 250 μM chloroform–solubilized cholesterol and kept for overnight evaporation. The thin lipid film of mixed lipids obtained after removal of residual chloroform was dissolved in 500 μl of 20 mM Tris pH 7.5 and 150 mM NaCl with gentle pipetting. The solution was next incubated at 55 °C for 30 min. This colloidal heated solution was subjected to extrusion as described above. The PC-Chol unilamellar vesicles were used for downstream experiments and the remaining was stored at 4 °C for not more than 3 days.

#### PA liposome

PA solution (preparation described in previous section) was incubated at 55 °C for 30 min. The heated solution was subjected to extrusion as mentioned above and used for liposome sedimentation assay.

#### Model membrane preparation

Powder form of Egg-PC (Sigma Aldrich), L-α-phosphatidylethanolamine (Sigma Aldrich), 16:0 Cardiolipin (Avanti Polar Lipids), 3-sn-PA (Sigma Aldrich), Brain PS (Avanti Polar Lipids) was dissolved in chloroform. Next, 270 μM PC, 145 μM phosphatidylethanolamine, 65 μM PIP4, 5 μM CL, 5 μM PA, 10 μM PS were mixed and kept for overnight evaporation. The thin lipid film obtained the following day after removal of residual chloroform was dissolved in 500 μl of 20 mM Tris pH 7.5 and 150 mM NaCl with gentle pipetting. The solution was next incubated at 55 °C for 30 min. The resultant colloidal heated solution was subjected to extrusion as mentioned above. Finally, the model membrane liposomes comprising a mixture of PC, phosphatidylethanolamine, PIP4, CL, PA, and PS were used for downstream experiments and the remaining was stored at 4 °C for not more than 3 days.

#### CF-engulfed liposomes

At first, a 20 mM CF stock solution (pH 7.4) was prepared. For largescale preparation of liposomes, chloroform-dissolved solution of 2.1 mM PC, 1.45 mM phosphatidylethanolamine, 0.52 mM PIP4, 0.04 mM CL, 0.04 mM PA, and 0.08 mM PS were mixed and kept for overnight evaporation. The resultant dried, thin lipid film was dissolved with gentle pipetting in 500 μl of 20 mM Tris pH 7.5 and 150 mM NaCl. To this solution, 500 μl of 20 mM CF solution was added and mixed by gentle pipetting. This was followed by incubation at 55 °C for 30 min. Next, the mixture was sonicated in a bath sonicator three times for 30 s with intermittent vortexing for 15 s. This was followed by extrusion according to steps mentioned above. Following extrusion, the liposomes that harbored CF were separated from free-CF by using a room-temperature gravity sizing column pre-packed with Sephadex G-25 resin. The CF liposomes were then stored at 4 °C and used the following day for membrane permeabilization assay.

### Liposome sedimentation assay

EspB_1–332_ heptamers (2 μM) were incubated with 50 μl of TCE liposomes (stock 1 mg/ml), 50 μl of PC-Chol liposomes (stock 500 μM), and 50 μl of model membrane preparation (stock 500 μM), in a reaction volume of 100 μl in 20 mM Tris (pH 7.5) and 150 mM NaCl for 30 min at RT. The reaction mixture was subjected to ultracentrifugation at 35,000 rpm for 45 min. After separating the supernatant, the pellet fraction was dissolved in 50 μl of 20 mM Tris (pH 7.5) and 150 mM NaCl. Equal volumes (20 μl) of the supernatant and the pellet fractions were analyzed by 12% SDS-PAGE/Coomassie staining.

For PA liposome-EspB_1–332_ sedimentation assay, 2 μM heptameric protein was incubated with serial dilutions of PA liposomes (highest ligand concentration 2 mM to lowest ligand concentration 2 μM) in a reaction volume of 100 μl in 20 mM Tris (pH 7.5) and 150 mM NaCl for 30 min at RT. This was followed by ultracentrifugation at 350,000 rpm for 45 min. The supernatants were transferred to fresh microcentrifuge tubes and the pellets were dissolved in 100 μl of 20 mM Tris (pH 7.5) and 150 mM NaCl. Equal volumes (20 μl) of the supernatant and the pellet fractions were analyzed by 12% SDS-PAGE/Coomassie staining.

### Nano-differential scanning fluorimetry thermal melt

Thermal unfolding of EspB_1–332_ monomers and heptamers in the presence and absence of lipids was carried out using a nanoDSF (Prometheus NT.48). Nearly 0.2 mg/ml EspB_1–332_ monomer (∼5 μM considering monomeric mass) and heptamer (∼700 nM considering heptameric mass) were incubated with 1 mg/ml PA and PS solutions (∼1 mM) respectively, for at least 15 mins at RT before analysis. As negative control to study lipid-dependent oligomerization of EspB_1–332_ monomers, 0.2 mg/ml EspB_1–332_ monomer (∼5 μM considering monomeric mass) were incubated with 1 mg/ml TCE lipid solution and 1 mg/ml PC lipid solution (∼1 mM PC), for at least 15 min at RT before analysis. Two independent sets of EspB_1–332_ purification were used for measurements in the presence or absence of lipids in a temperature range of 20 to 95 °C at 50% LED power and initial discovery scan counts (350 nm) ranging between 6000 and 10,000. The same amount of PA, PS, TCE, and PC were separately analyzed to record the thermal melt spectra of only lipids.

### Microscale thermophoresis

Heptameric EspB_1–332_ and phospholipid interaction assay was done using MST (Nanotemper) (48). Freshly purified heptameric fraction of EspB_1–332_ was labeled with RED-NHS second generation amine reactive dye equimolar ratio. Monolith NT.115 standard MST capillaries were used in each experiment. In binding reaction, the labeled protein concentration was kept constant at approximately 20 nM. The protein was incubated with 16 two-fold serial dilutions of ligands dissolved in 20 mM Tris (pH 7.5), 150 mM NaCl. For PA, PS, PIP4, CL, PC, and phosphatidylethanolamine, the highest ligand concentration was 500 μM. The protein and lipids were both diluted in 20 mM Tris (pH 7.5), 150 mM NaCl, and 0.05% Tween-20. The assays were performed with two independent set of purifications and each time, binding with PA, PS, and PIP4 was obtained in micromolar range. The samples were analyzed with a Monolith NT.115 pico device using MO.Control Software (https://nanotempertech.com/monolith-mo-control-software/; Nanotemper). Binding was measured with a Monolith NT.115 pico device using MO.Control Software (Nanotemper) at RT (LED/excitation power setting 20%, MST power setting 40%). Data were analyzed using MO.Affinity Analysis software (https://shop.nanotempertech.com/en/moaffinity-analysis-software-unlimited-licenses-34; version 2.2.5, NanoTemper Technologies) at different standard MST-off times.

### Liposome leakage assay

The CF liposomes were incubated at RT with 1 μM freshly purified heptameric EspB_1–332_ protein, and the continuous fluorescence was recorded for 30 min. For prolonged incubation, single time point fluorescence was recorded for 0 h, 6 h, 12 h, and 24 h. As control to signify 100% lysis, 0.1% Triton-X was added to the CF liposomes. Experiment was performed with two independent set of purifications.

### NS-TEM sample preparation

#### TEM analysis of purified EspB_1–332_

For each peak fraction (9 ml, 11.4 ml, and 13.8 ml), approximately 3.5 μl of 0.1 mg/ml protein was added on 400-mesh Cu TEM grids which were freshly glow-discharged (negative polarity) for 30 s in GloQube glow-discharge system. The sample was incubated at RT for 1 min. The surplus solution was then carefully blotted off using Whatman filter paper. This was followed by negative staining using 1% freshly prepared uranyl-acetate solution.

#### TEM analysis of heptameric EspB_1–332_ with TCE liposomes, PC-Chol liposomes

Approximately, 1 mg/ml EspB_1–332_ heptamers were incubated with TCE and PC-Chol liposomes at RT for 20 min. The reaction mixtures were then subjected to centrifugation at 13,000 rpm for 30 min (liposome sedimentation assay). The supernatant was separated from the pellet. The pellet comprising the bulk of the liposomes were dissolved in 20 mM Tris (pH 7.5), 150 mM NaCl buffer such that the volume was equal to that of the supernatant. The supernatants (diluted to ∼0.1 mg/ml) and the pellet solutions (undiluted) were negatively stained as described above.

#### TEM analysis of heptameric EspB_1–332_ with PA and PS

Approximately, 1 mg/ml EspB_1–332_ heptamers were incubated with 1 mM PA and PS solutions, respectively, and incubated at RT for 20 min. This was followed by negative staining as described above.

#### TEM analysis of monomeric EspB_1–332_ with PA and PS

Approximately, 0.1 mg/ml EspB_1–332_ monomers were incubated with 1 mM PA and PS solutions, respectively, and incubated at RT for 20 min. This was followed by negative staining as described above.

#### TEM analysis of heptameric EspB_1–332_ with model membrane

Approximately, 0.5 mg/ml EspB_1–332_ heptamers were incubated with model membrane solution and incubated at RT for 20 min. This was followed by negative staining as described above.

#### TEM analysis of heptameric EspB_1–332_ with yeast mitochondria

*Saccharomyces cerevisiae* mitochondria was a kind gift from the laboratory of Prof. Patrick D' Silva, Indian Institute of Science, Bangalore. Approximately, 0.5 mg/ml EspB_1–332_ heptamers were incubated with 50 ng/μl yeast mitochondria at RT for 20 min. This was followed by negative staining as described above.

The TEM data were acquired on a 120 kV Talos L120C RT electron microscope equipped with a bottom-mounted Ceta camera (4K × 4K) at different magnifications ranging between 43,000× and 120,000× magnification between calibrated pixel size of 3.68 to 0.92 Å/pixel at specimen level.

### NS-TEM data processing

The NS-TEM raw micrographs were imported into EMAN 2.1 (49), and the best micrographs were retained for calculating the reference-free 2D class averages. Around 3000 particles were manually picked for EspB_1–332_ fused rings, EspB_1–332_ heptamers, EspB_1–332_ monomers using EMAN 2.1, and particles were extracted using e2boxer.py in EMAN 2.1. The extracted particles were imported into RELION 3.0 (50) and 2–3 rounds of reference-free 2D class averages were calculated to classify the particles to increase the signal to noise ratio. The class averages with prominent features were selected for EspB_1–332_ fused rings, EspB_1–332_ heptamers, EspB_1–332_ monomer samples, respectively. The cleaned particle sets thus obtained after the initial rounds of 2D classification were reclassified using simple_prime2D of SIMPLE 2.0 (51).

### Cryo-EM sample preparation and data collection

The EspB_1–332_ heptamer protein sample was taken for cryo-EM to study with and without PA. Initially, 300 mesh copper Quantifoil R 1.2/1.3 grids were glow discharged in GloQube glow discharge apparatus at 20 mA for 90 s. For control EspB_1–332_ heptamers, protein was concentrated to 6 mg/ml and 0.03% FOM was added to the sample immediately before application on cryo-TEM grid. Three microliters of the FOM-EspB_1–332_ sample was applied to the glow discharged grids and incubated at 100% humidity for 10 s. Excess sample was blotted for 7 s at −2 blot force.

In the case of EspB_1–332_ heptamers in the presence of PA, nearly 1 mg/ml protein was incubated with 2 mM PA at RT for 20 min. Three microliters of the PA-EspB_1–332_ sample were applied on the glow discharged grids and incubated at 100% humidity for 10 s. Excess sample was blotted for 7 s at 0 blot force.

In the case of EspB_1–332_ monomers in the presence of PA, nearly 0.2 mg/ml protein was incubated with 1 mM PA at RT for 20 min. Three microliters of the PA-EspB_1–332_ monomer sample were applied on the glow discharged grids and incubated at 100% humidity for 10 s. Excess sample was blotted for 7 s at 0 blot force.

This was followed by plunge-freezing in liquid ethane cooled by ambient liquid nitrogen in FEI Vitrobot Mark IV plunger. Imaging was performed in a Thermo Fisher Scientific 200 kV Talos Arctica Transmission Electron Microscope equipped with K2 Summit Direct Electron Detector (4K × 4K) (Gatan Inc). Data collection was performed using automated data collecting software package, LatitudeS (52) (Gatan Inc) at 54,000× magnification at a calibrated pixel size of 0.92 Å/pixel at specimen level. Movies were recorded for 40 frames and 8 s with a total calibrated dose of 60 e^−^/Å^2^. The defocus range for data collection ranged from −0.75 μm to −2.5 μm.

### Cryo-EM data processing

For both the cryo-EM datasets, the same steps were followed as mentioned next. A total of 3000 movies were first corrected for beam-induced motion using RELION 3.1 implementation of MotionCor2 (53). The resultant motion-corrected micrographs were imported into cisTEM (54) to sort the data based on the signal to noise ratio (removing carbon areas, crystalline ice, beam-damaged images) and to select micrographs with signal better than 7 Å resolution. The contrast transfer function of the selected micrographs was estimated using CTFFIND 4.1.13 (55). A small subset of particles were manually picked in RELION 3.1 to generate the reference-free 2D class averages. The best 2D classes were selected to use as a template for automated particle picking.

### Control EspB_1–332_ data processing pipeline

Approximately, 1,130,382 particles were picked and extracted at a box size of 240 pixels. After two rounds of reference-free 2D classification, 558,582 particles were obtained. These curated particles were next subjected to a round of 3D classification using C7 symmetry. 40 Å low-pass filter was applied on 6xzc.pdb, and the resultant featureless map was used as initial model for 3D classification. The best class showing the highest resolution and distinct structural features was selected for autorefinement with C7 symmetry. The contrast transfer function of the refined particle set was further improved using beamtilt estimation and owing to the high resolution features of the structure, this was followed by particle polishing and one round of autorefinement using C7 symmetry. The final cryo-EM density map of control EspB_1–332_ heptamer was obtained at 4.5 Å at 0.143 Fourier Shell Correlation.

### PA-EspB_1–332_ data processing pipeline

Approximately, 751,763 particles were picked and extracted at a box size of 240 pixels. After two rounds of reference-free 2D classification, 300,140 particles were obtained. These curated particles were next subjected to a round of 3D classification using C7 symmetry. 40 Å low-pass filter was applied on 6xzc.pdb, and the resultant featureless map was used as initial model for 3D classification. The class with high resolution structural feature was selected for autorefinement using C7 symmetry. Beamtilt estimation of the refined particle set did not further improve the map resolution. The final cryo-EM density map of PA-EspB_1-332_ heptamer was obtained at 6.6 Å at 0.143 FSC.

All visualizations were performed using UCSF Chimera (56) and UCSF ChimeraX (57).

## Data availability

The EM density maps have been deposited to the Electron Microscopy Data Bank (EMDB) with the accession codes EMD-34878 (for EspB_1–332_ map) and EMD-33752 (for EspB_1–332_ with PA map).

All other data are contained within the manuscript.

## Supporting information

This article contains supporting information.

## Conflict of interest

The authors declare no conflict of interest with the contents of this article.
