# Supplementary material for: Cryo-EM reveals the membrane-binding phenomenon of EspB, a virulence factor of the mycobacterial type VII secretion system
Source: J Biol Chem. 2023 Mar 6;299(4):104589. doi: 10.1016/j.jbc.2023.104589 (PMC10140165; doi:10.1016/j.jbc.2023.104589)
Supplement: Supporting Figures S1–S14 [file mmc1.pdf]

Supporting Information

Cryo-EM reveals the membrane-binding phenomenon of EspB, a virulence factor of the mycobacterial type VII secretion system

Nayanika Sengupta, Surekha Padmanaban, and Somnath Dutta*

Molecular Biophysics Unit, Indian Institute of Science, Bangalore 560012, India

Tel.: 080-22933453; Fax: 080-2360 0535

*Correspondence: somnath@iisc.ac.in (S.D.)

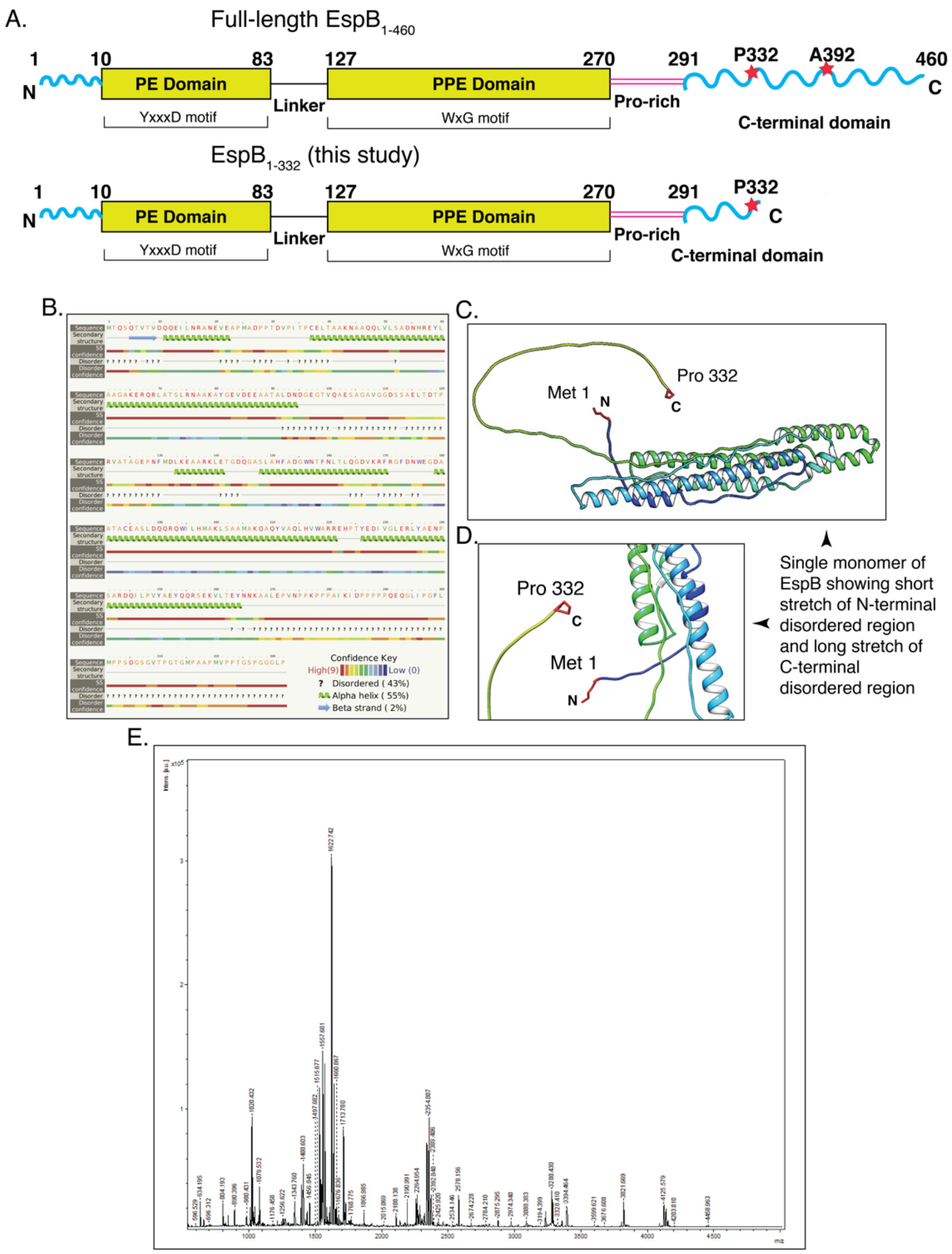

Supplementary Figure 1: (A) Schematic representation of the different domains harbored in full-length $EspB_{1-460}$ and in $EspB_{1-332}$ used in this study. Blue curved lines represent areas of disorder, rectangular boxes represent the folded PE and PPE domains, magenta-colored parallel lines show the proline rich region that links the N-terminal domain to the C-terminal domain. Red stars denote $MycP_1$ cleavage sites. (B) Secondary structure prediction of $EspB_{1-332}$, using Phyre2 showing a majorly α-helical N-terminal domain and a disordered C-terminal domain. (C) Alpha fold representation of monomeric $EspB_{1-332}$ denoting the relative orientation of the N-terminal Met and C-terminal Pro. (D) Enlarged view of the flexible terminal of $EspB_{1-332}$ reveals a short N-terminal disorder stretch and a nearly ~50 residue long disordered C-terminal. (E) MALDI-TOF spectrum of recombinant $EspB_{1-332}$.

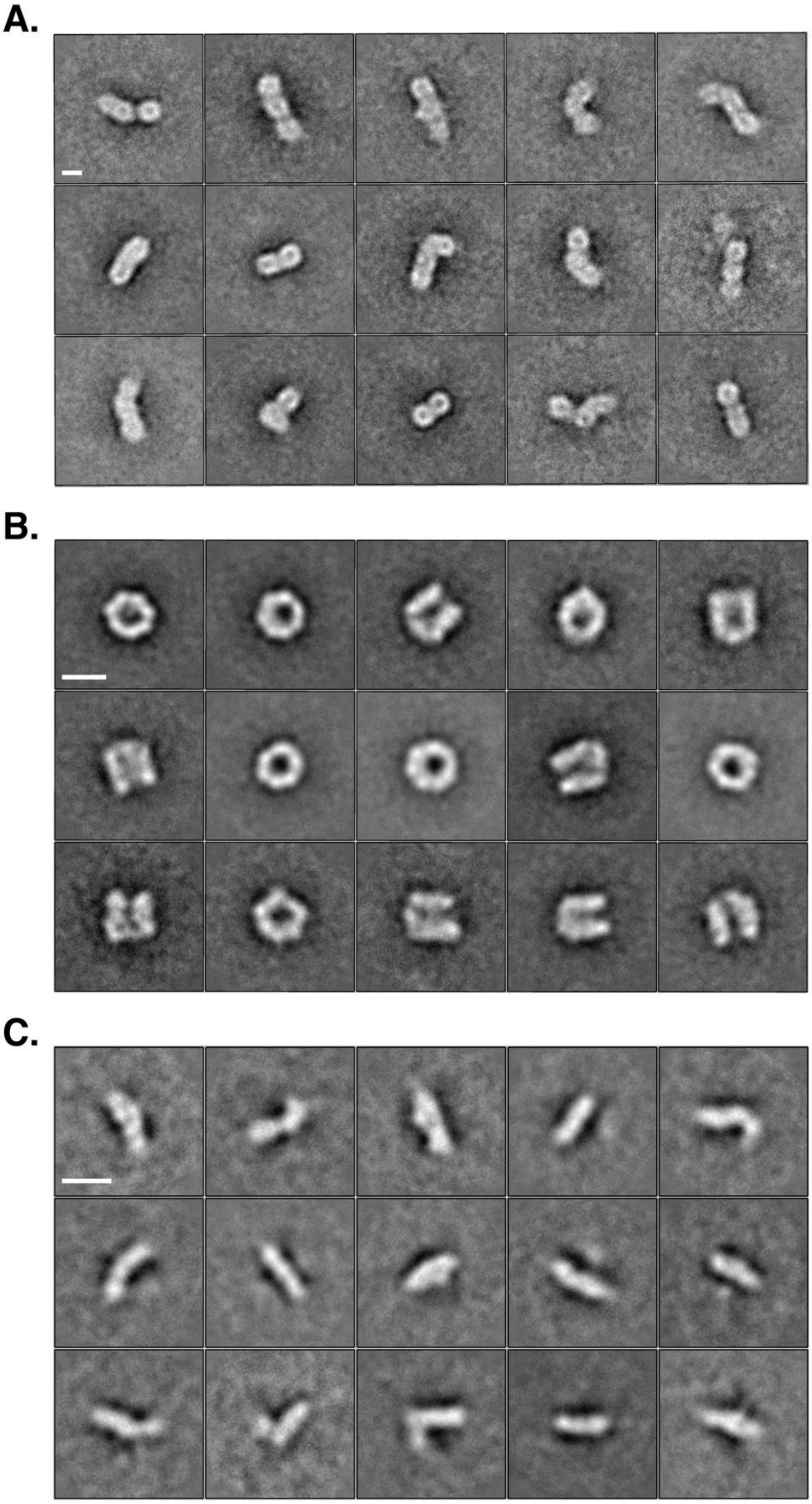

Supplementary Figure 2: **Negative staining transmission electron microscopy (NS-TEM) 2D class averages.** (A) Extended 2D classes of the higher order oligomeric fraction of $EspB_{1-332}$, selected views of which have been listed in Figure 1D. (B) Extended 2D classes of the discrete ring-like population of $EspB_{1-332}$, selected views of which have been listed in Figure 1D. (C) Extended 2D classes of the open chain-like population of $EspB_{1-332}$, selected views of which have been listed in Figure 1D. Scale bars denote 10 nm.

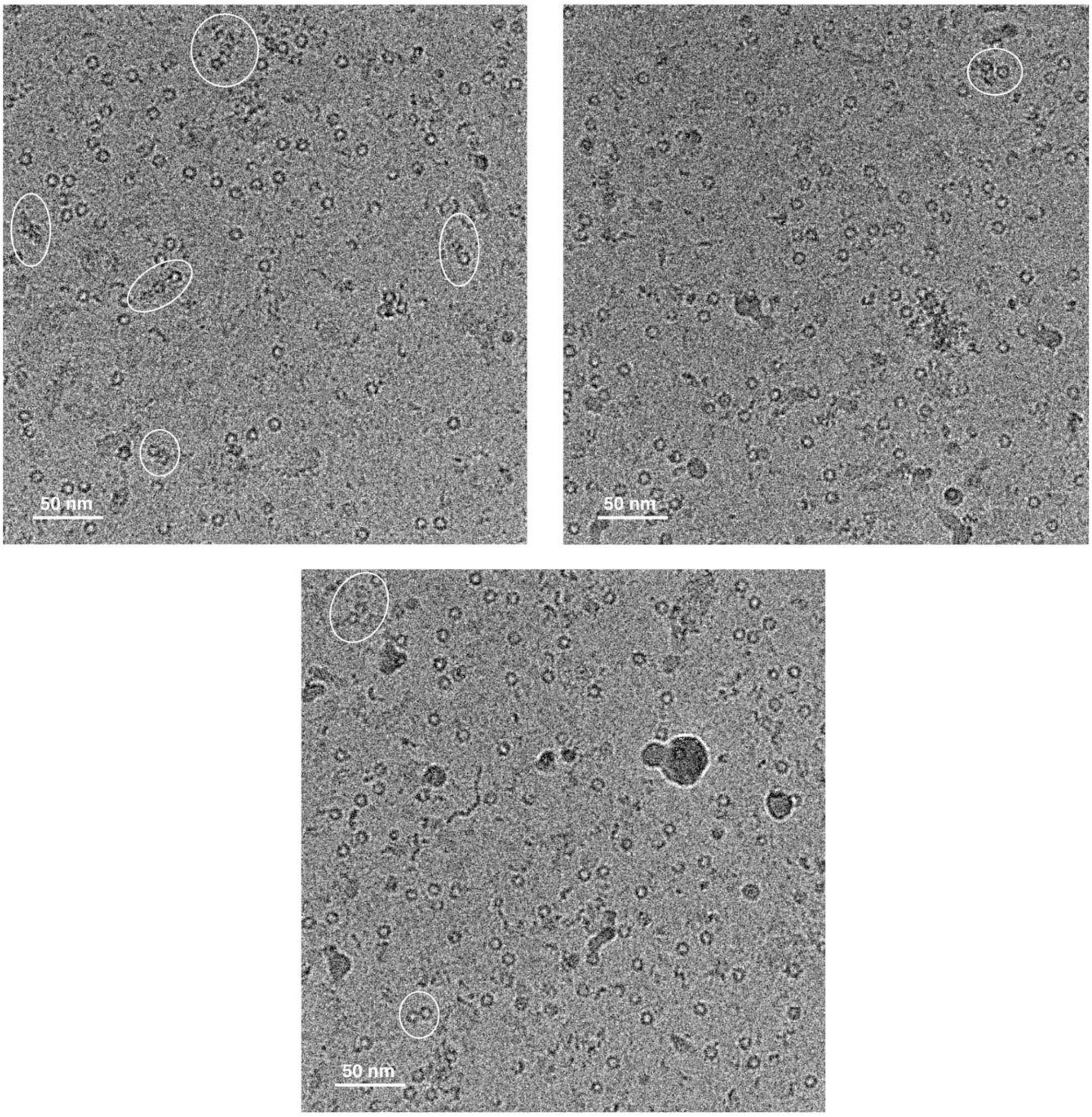

Supplementary Figure 3: **Different areas of the cryo-EM grid reveals the instability of the fused EspB$_{1-332}$ rings in cryogenic conditions.** Major population of the multimers as observed in NS-TEM (Figure 1D) appear to be separated into individual hexameric or heptameric rings. Few conjoined oligomers coexist with ring-like oligomer and have been demarcated within white boundaries.

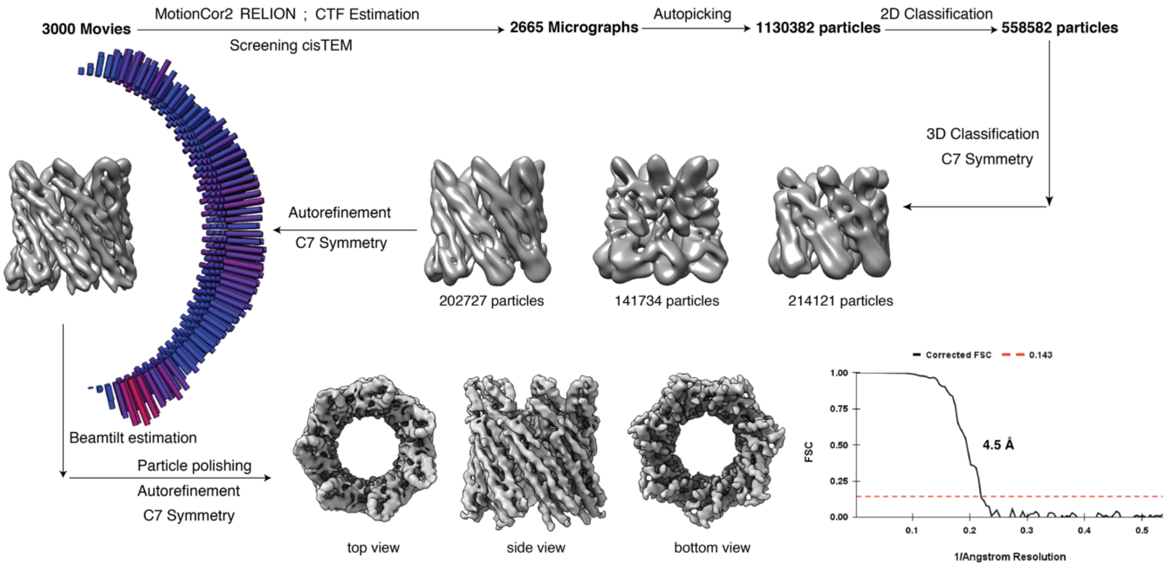

Supplementary Figure 4: Cryo-EM data processing pipeline followed for control $EspB_{1\text{-}332}$ dataset. Gold standard Fourier Shell Correlation (FSC) calculation shows a resolution of 4.5 Å. See Methods for extended procedures.

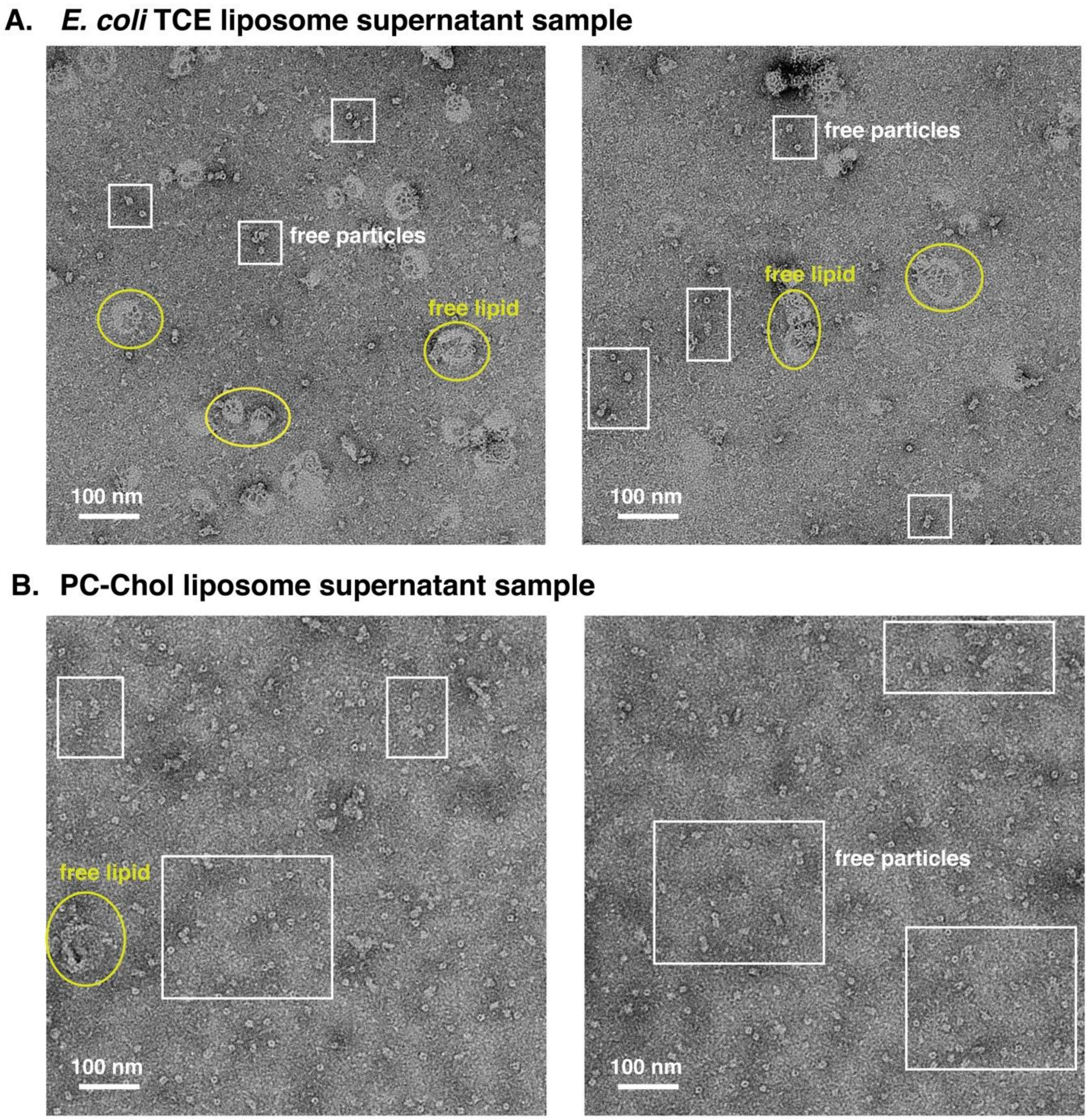

Supplementary Figure 5: **NS-TEM analysis of the supernatant fractions obtained after liposome sedimentation assay.** (A) *E. coli* TCE liposome treated $EspB_{1\text{-}332}$ predominantly segregates into the supernatant as compared to the pellet (Figure 3A). Proteins appear isolated from the light stained areas which possibly denote lipid patches. (B) PC-Chol liposome treated $EspB_{1\text{-}332}$ predominantly segregates into the supernatant as compared to the pellet (Figure 3A). White boxed areas show free particles of protein whereas the yellow elliptical boundaries demarcate the free lipids.

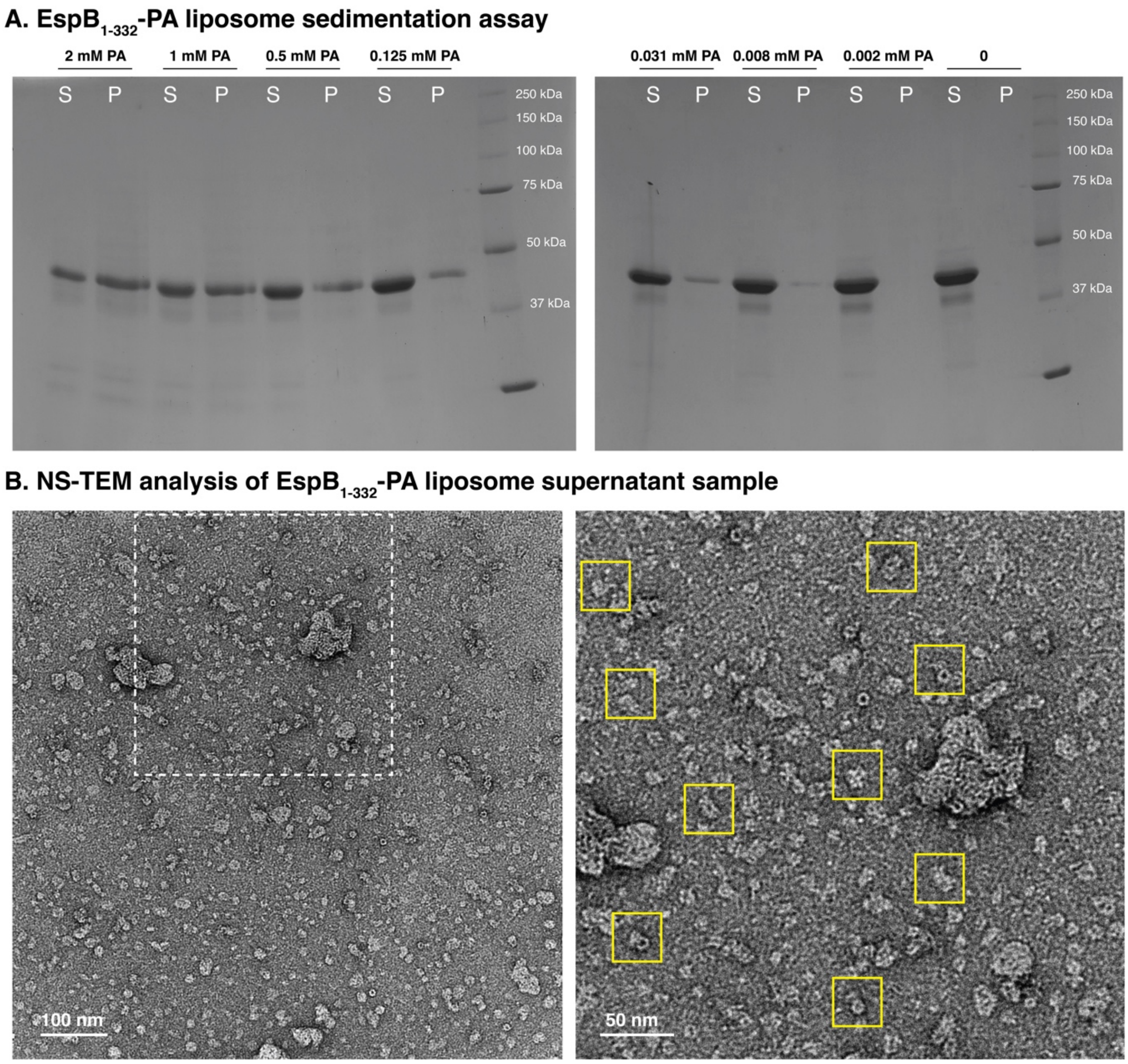

Supplementary Figure 6: **Liposome sedimentation assay to study EspB$_{1\text{-}332}$-PA interaction using serials of PA liposome concentration.** (A) 12% SDS-PAGE gels showing pellet and supernatant fractions obtained after performing PA liposome sedimentation assay with 2 µM EspB$_{1\text{-}332}$ heptamers with serial dilutions of PA liposomes. Protein starts appearing in the pellet fraction with PA liposome concentrations as low as 8 µM (right). More than half of the protein sediments near PA concentration ~ 2 mM (left). (B) NS-TEM analysis of the supernatant fraction obtained with highest concentration of PA liposome (2 mM) shows the presence of smaller PA assemblies. Representative raw micrograph has been enlarged on the right and yellow boxes have been used to highlight top and side views of EspB$_{1\text{-}332}$, which appear to be connected to extra densities, possibly corresponding to PA lipid.

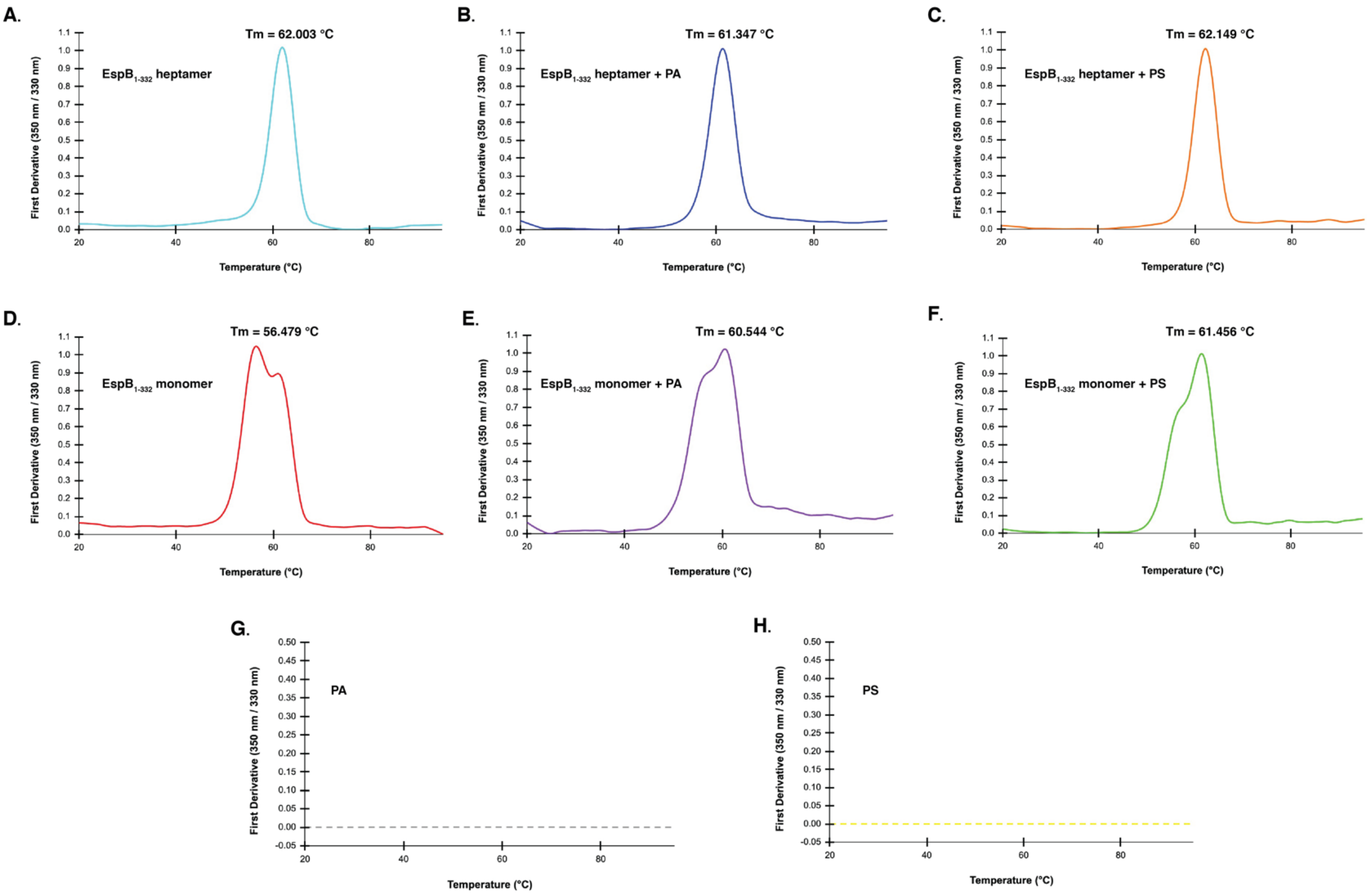

Supplementary Figure 7: **Thermal melt profile of $EspB_{1\text{-}332}$ with and without PA and PS.** (A) Thermal stability of $EspB_{1\text{-}332}$ heptamer (Figure 4A). (B) Thermal stability of $EspB_{1\text{-}332}$ heptamer in presence of PA (4A). (C) Thermal stability of $EspB_{1\text{-}332}$ heptamer in presence of PS (Figure 4A). (D) Thermal stability of $EspB_{1\text{-}332}$ monomer (Figure 4A). (E) Thermal stability of $EspB_{1\text{-}332}$ monomer in presence of PA (Figure 4A). (F) Thermal stability of $EspB_{1\text{-}332}$ monomer in presence of PS (Figure 4A). (G) Melting scan of PA (Figure 4A). (H) Melting scan of PS (Figure 4A).

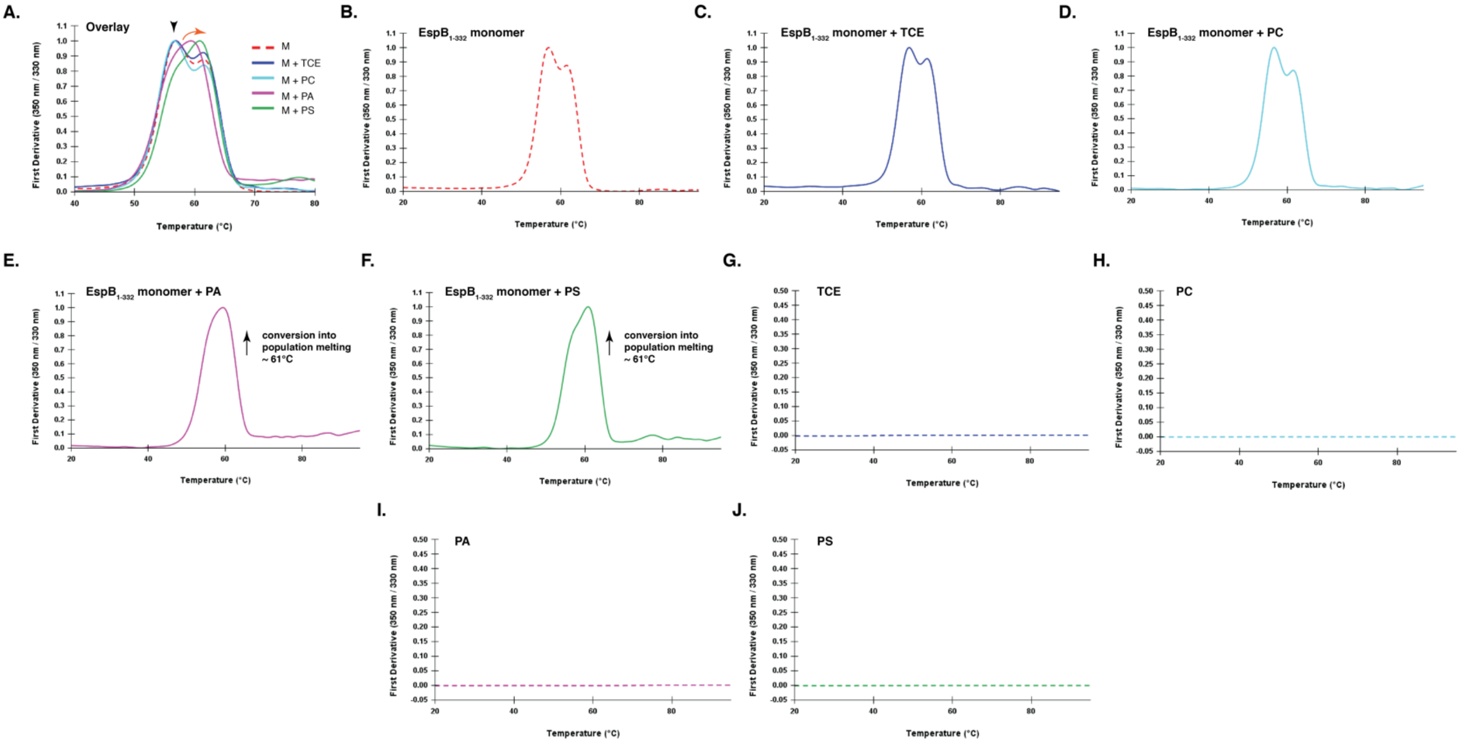

Supplementary Figure 8: **Effect of different lipids on the thermal melt profile of EspB$_{1\text{-}332}$ monomers.** (A) Overlay of the melting scan profiles of EspB$_{1\text{-}332}$ monomer in the absence and presence of different lipids. Black arrowhead has been used to denote that the proportion of protein with Tm ~ 56 °C remains constant in the case of only EspB$_{1\text{-}332}$ monomers and when incubated with *E. coli* total cell extract (TCE) lipid and phosphatidylcholine (PC). Salmon curved arrow has been used to show the shift in the population towards a higher Tm of ~ 61°C, in the presence of PA and PS. (B) Melting scan of EspB$_{1\text{-}332}$ monomer. (C) Melting scan of EspB$_{1\text{-}332}$ monomer in presence of TCE. (D) Melting scan of EspB$_{1\text{-}332}$ monomer in presence of PC. (E) Melting scan of EspB$_{1\text{-}332}$ monomer in presence of PA. (F) Melting scan of EspB$_{1\text{-}332}$ monomer in presence of PS. (G) Melting scan of TCE. (H) Melting scan of PC. (I) Melting scan of PA. (J) Melting scan of PS.

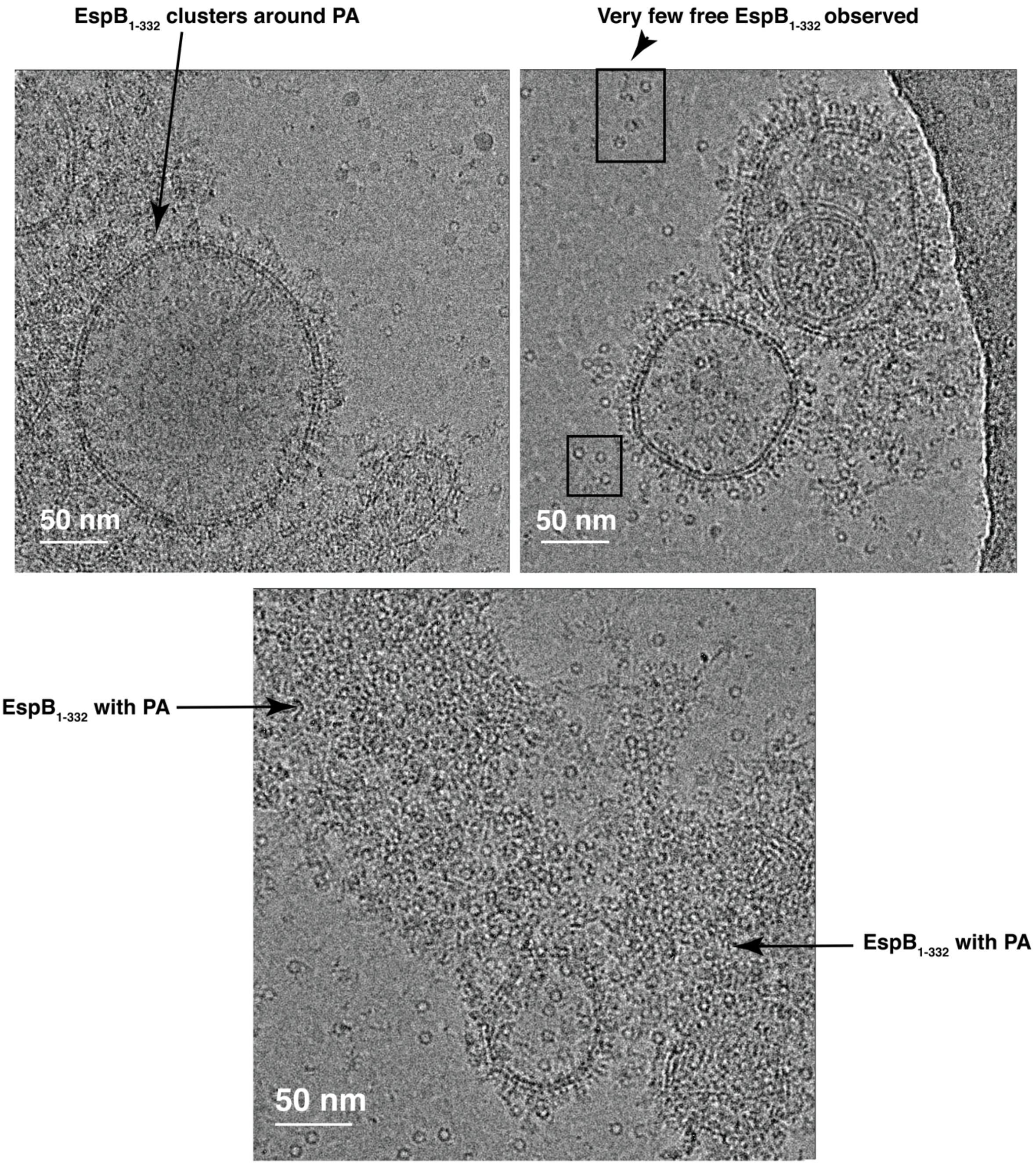

Supplementary Figure 9: **A montage of cryo-EM raw micrographs that show a remarkable affinity of EspB$_{1-332}$ towards PA vesicles.** Only few protein particles appear in the background while most of the EspB$_{1-332}$ cluster around PA vesicles.

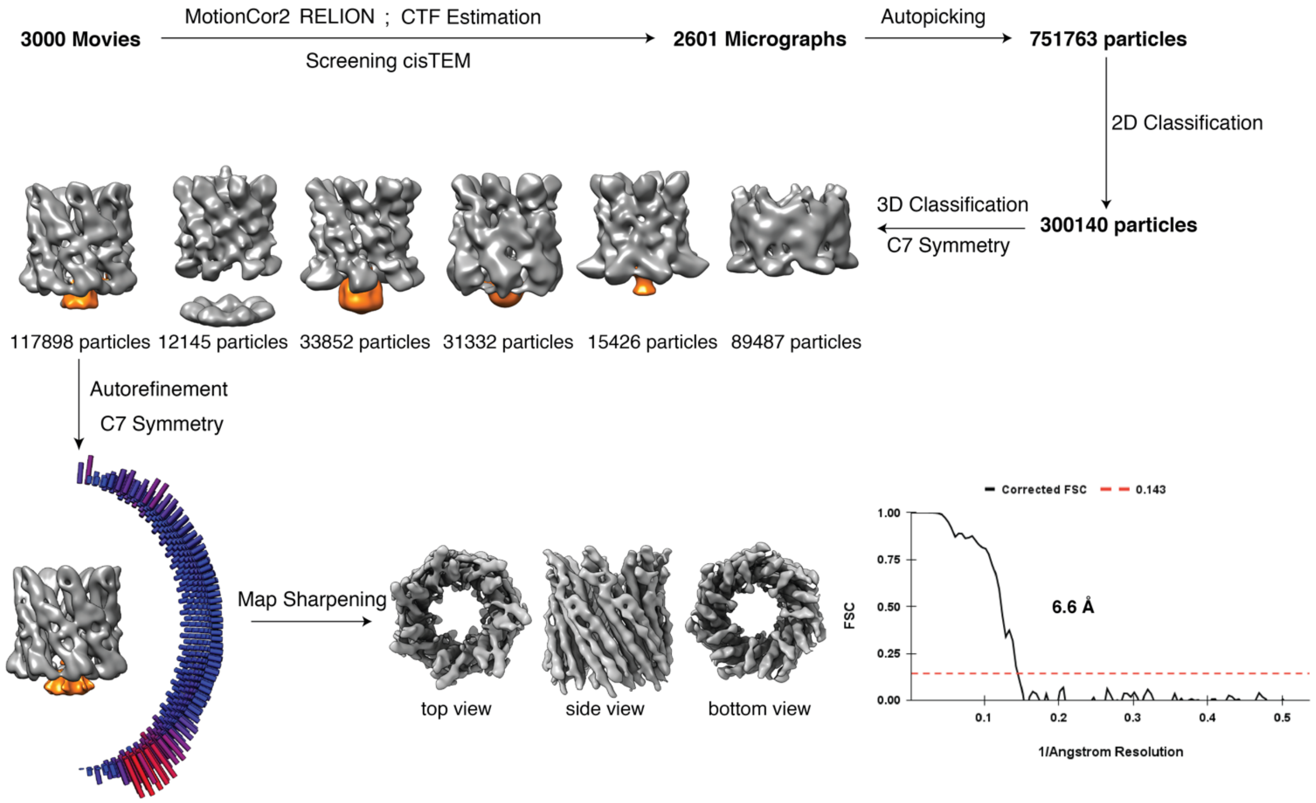

Supplementary Figure 10: Cryo-EM data processing pipeline followed for PA-EspB$_{1\text{-}332}$ dataset. Orange color has been used to highlight the additional density obtained at the bottom of the map. Gold standard Fourier Shell Correlation (FSC) calculation shows a resolution of 6.6 Å. See Methods for extended procedures.

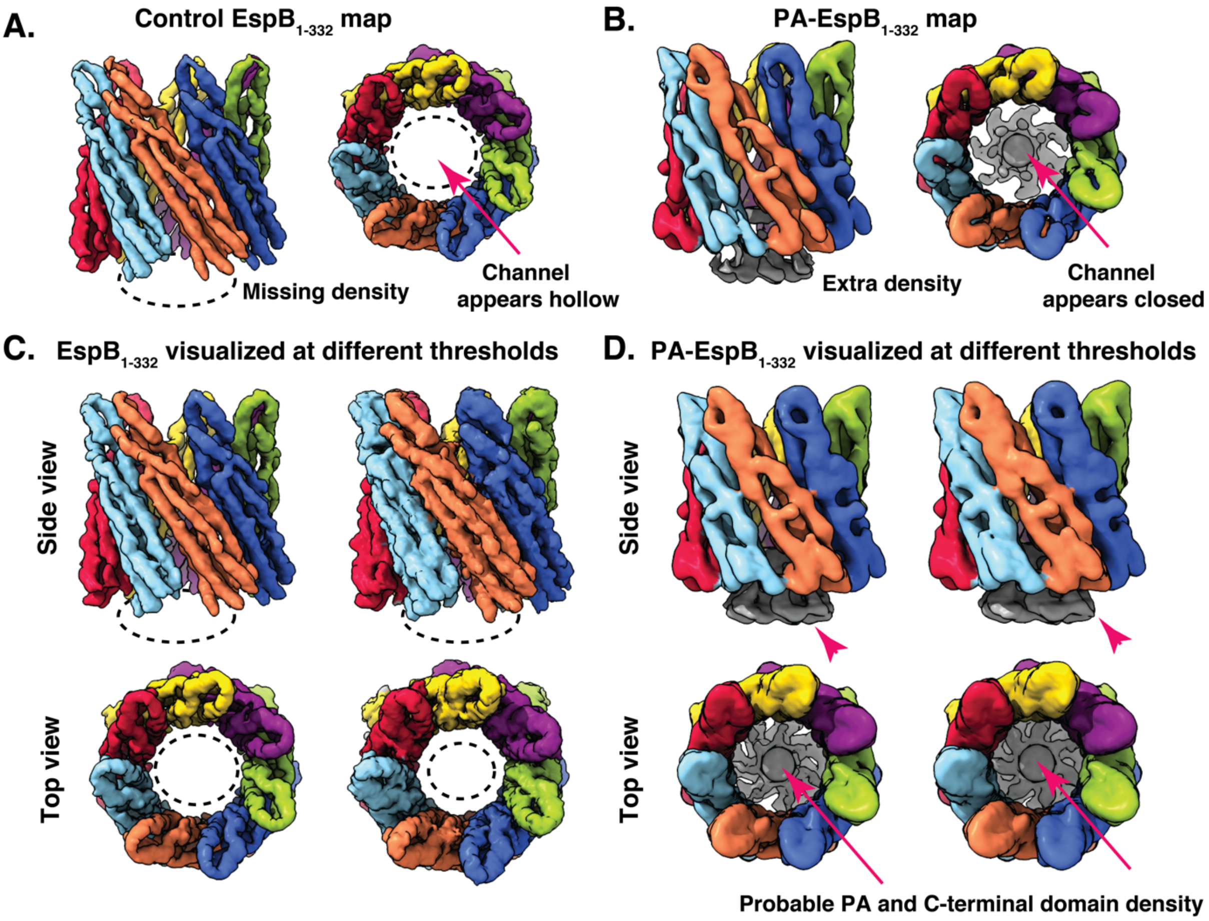

Supplementary Figure 11: Comparative analysis of EspB$_{1\text{-}332}$ structures obtained without and with PA. (A) Side and top views of DeepEMhancer map of control EspB$_{1\text{-}332}$ shows only the N-terminal domain. (B) Side and top views of DeepEMhancer map of PA-EspB$_{1\text{-}332}$ shows a firm density at one end of the channel, along with the N-terminal domain. (C) Boosting the volume threshold of control map does not make the additional density appear. Upper panel shows the volume boosted side views whereas the bottom panels represent the corresponding top views. (D) Boosting the volume threshold of DeepEMhancer sharpened PA treated map shows an increase in the volume of the additional density. Upper panel shows the volume boosted side views whereas the bottom panels represent the corresponding top views.

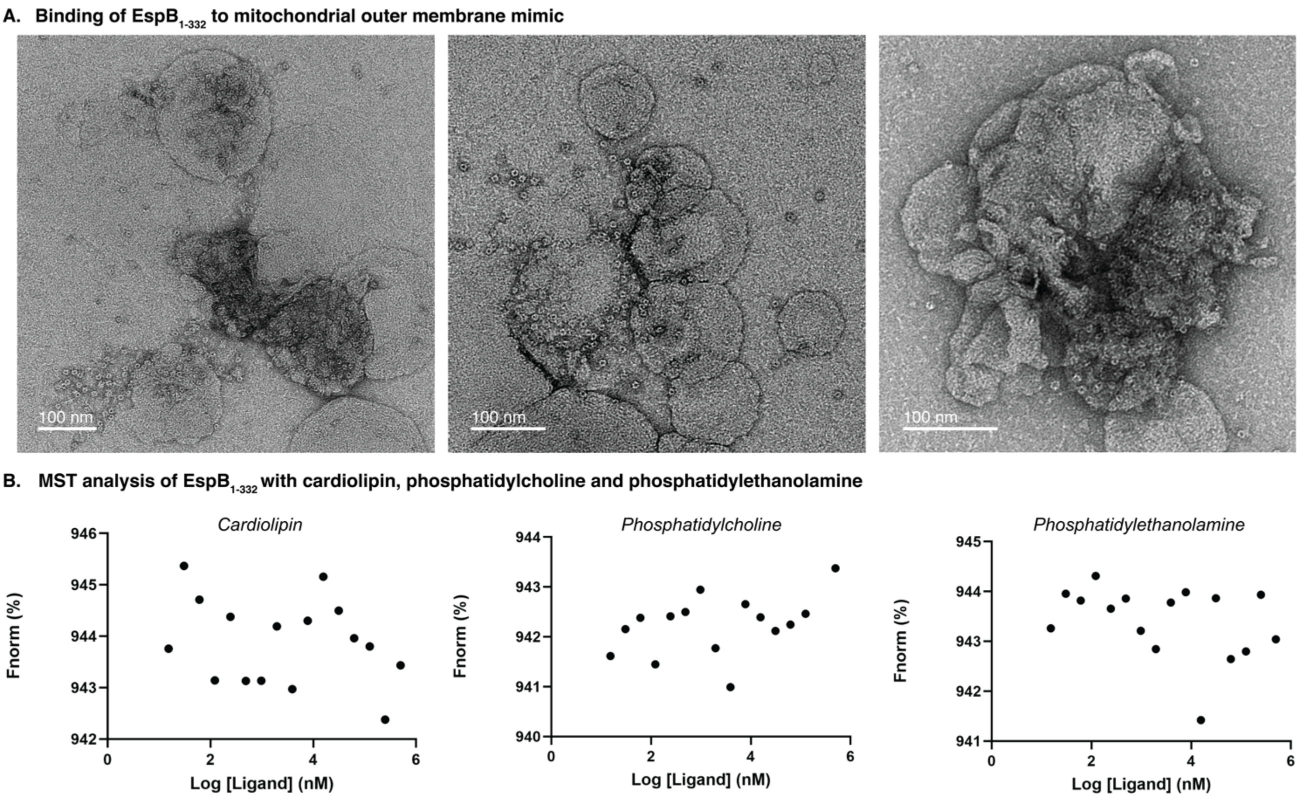

Supplementary Figure 12: (A) Collection of NS-TEM images showing the affinity of $EspB_{1-332}$ heptamers with model membrane. (B) Failure to observe binding affinity with cardiolipin, phosphatidylcholine and phosphatidylethanolamine, respectively.

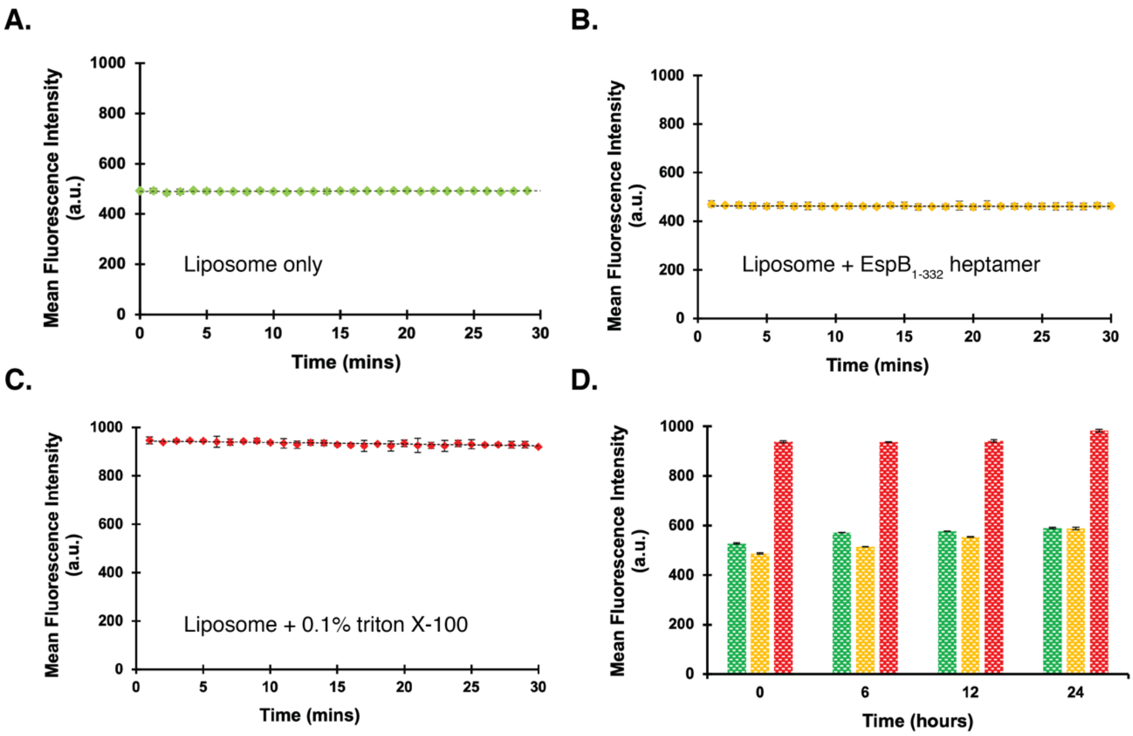

Supplementary Figure 13: (A) Carboxyfluorescein leakage monitored over a period of 30 mins for model liposome. (B) Carboxyfluorescein leakage monitored over a period of 30 mins for model liposome incubated with $EspB_{1-332}$ heptamers. (C) Carboxyfluorescein leakage monitored over a period of 30 mins for model liposome treated with 0.1% triton X-100. (D) Bar diagram showing the comparative leakage of only liposomes (green), liposomes incubated with $EspB_{1-332}$ heptamers (yellow) and liposomes treated with 0.1% triton X-100 (red), over a period of 24 hours. Data presented here are mean ± SD from two independent sets of protein purification.

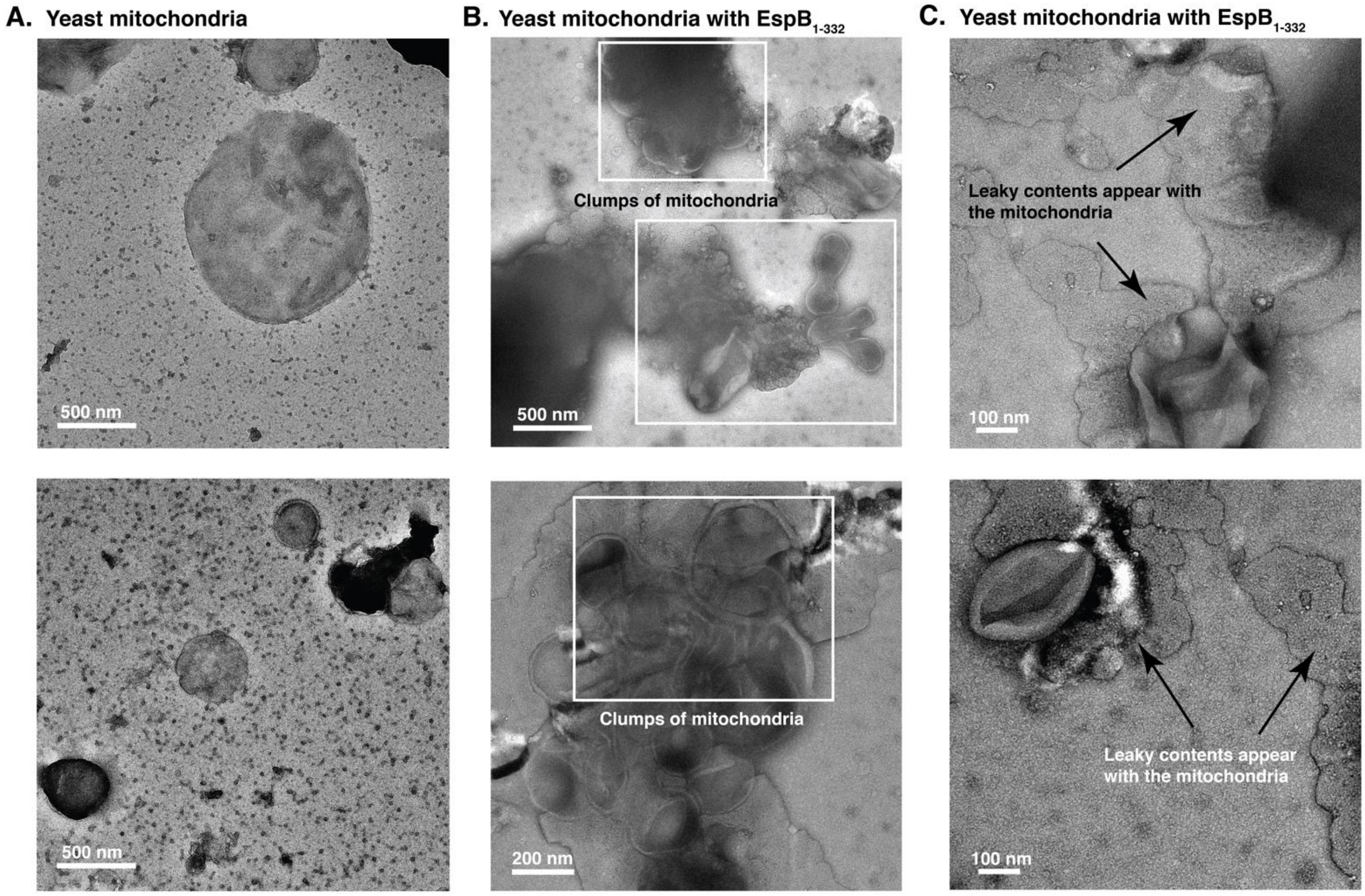

Supplementary Figure 14: **NS-TEM visualization of yeast mitochondria without and with EspB$_{1\text{-}332}$.** (A) Control mitochondria observed in different areas. (B) Altered morphology of mitochondria post incubation with EspB$_{1\text{-}332}$, indicating clumping of mitochondria. (C) shows dense patterns surrounding mitochondria giving an impression of leakage of organelle contents.
